# Rp-Bp and the Ribotools box for translatome profiling

**DOI:** 10.1093/nargab/lqag130

**Published:** 2026-11-03

**Authors:** Etienne Boileau, Maja Bencun, Francesca Tuorto, Isabel S Naarmann-de Vries, Pauline Fahjen, Philipp Mertins, Christoph Dieterich

**Affiliations:** Department of Internal Medicine III (Cardiology, Angiology, and Pneumology), University Hospital Heidelberg, Im Neuenheimer Feld 669, 69120 Heidelberg, Germany; Klaus Tschira Institute for Integrative Computational Cardiology, University of Heidelberg, Im Neuenheimer Feld 669, 69120 Heidelberg, Germany; German Center for Cardiovascular Research (DZHK) – Partner site Heidelberg/Mannheim, Im Neuenheimer Feld 410, 69120 Heidelberg, Germany; Department of Internal Medicine III (Cardiology, Angiology, and Pneumology), University Hospital Heidelberg, Im Neuenheimer Feld 669, 69120 Heidelberg, Germany; Klaus Tschira Institute for Integrative Computational Cardiology, University of Heidelberg, Im Neuenheimer Feld 669, 69120 Heidelberg, Germany; German Center for Cardiovascular Research (DZHK) – Partner site Heidelberg/Mannheim, Im Neuenheimer Feld 410, 69120 Heidelberg, Germany; Division of Biochemistry, Mannheim Institute for Innate Immunoscience (MI3), Mannheim Cancer Center, Medical Faculty Mannheim, Heidelberg University, Franz-Volhard-Str. 6, 68167 Mannheim, Germany; Center for Molecular Biology of Heidelberg University (ZMBH), DKFZ-ZMBH Alliance, 69120 Heidelberg, Germany; Department of Internal Medicine III (Cardiology, Angiology, and Pneumology), University Hospital Heidelberg, Im Neuenheimer Feld 669, 69120 Heidelberg, Germany; Klaus Tschira Institute for Integrative Computational Cardiology, University of Heidelberg, Im Neuenheimer Feld 669, 69120 Heidelberg, Germany; German Center for Cardiovascular Research (DZHK) – Partner site Heidelberg/Mannheim, Im Neuenheimer Feld 410, 69120 Heidelberg, Germany; Max Delbrück Center for Molecular Medicine (MDC), Robert-Rössle-Straße 10, 13125 Berlin, Germany; Max Delbrück Center for Molecular Medicine (MDC), Robert-Rössle-Straße 10, 13125 Berlin, Germany; Berlin Institute of Health at Charité – Universitätsmedizin Berlin, Charitéplatz 1, 10117 Berlin, Germany; Department of Internal Medicine III (Cardiology, Angiology, and Pneumology), University Hospital Heidelberg, Im Neuenheimer Feld 669, 69120 Heidelberg, Germany; Klaus Tschira Institute for Integrative Computational Cardiology, University of Heidelberg, Im Neuenheimer Feld 669, 69120 Heidelberg, Germany; German Center for Cardiovascular Research (DZHK) – Partner site Heidelberg/Mannheim, Im Neuenheimer Feld 410, 69120 Heidelberg, Germany

## Abstract

We present the Ribotools “box” for the analysis of matched ribosome profiling (Ribo-seq) and RNA sequencing data. Ribotools can be used in standalone mode or in combination with Rp-Bp for Ribo-seq quality control, open reading frames discovery, translation efficiency, and differential expression analyses. In this work, we use Ribotools to profile the differentiation of human induced pluripotent stem cells (hiPSCs) into cardiomyocytes to reveal a remarkably coherent transition from plastic, signaling-driven to stable, structurally committed cardiac cells, staged through multiple layers of translational control. Together with complementary proteomics, the resulting dataset provides a rich multi-omics resource for investigating the hiPSC model of cardiac lineage commitment. Additionally, we present a reproducible and extensible pipeline for benchmarking translation efficiency analysis tools. Ribotools and Rp-Bp are available on Bioconda and help to make research accessible and reproducible. Detailed documentation, test datasets, and tutorials are available at https://ribotools.readthedocs.io and https://rp-bp.readthedocs.io.

## Graphical Abstract

**Graphical Abstract:**
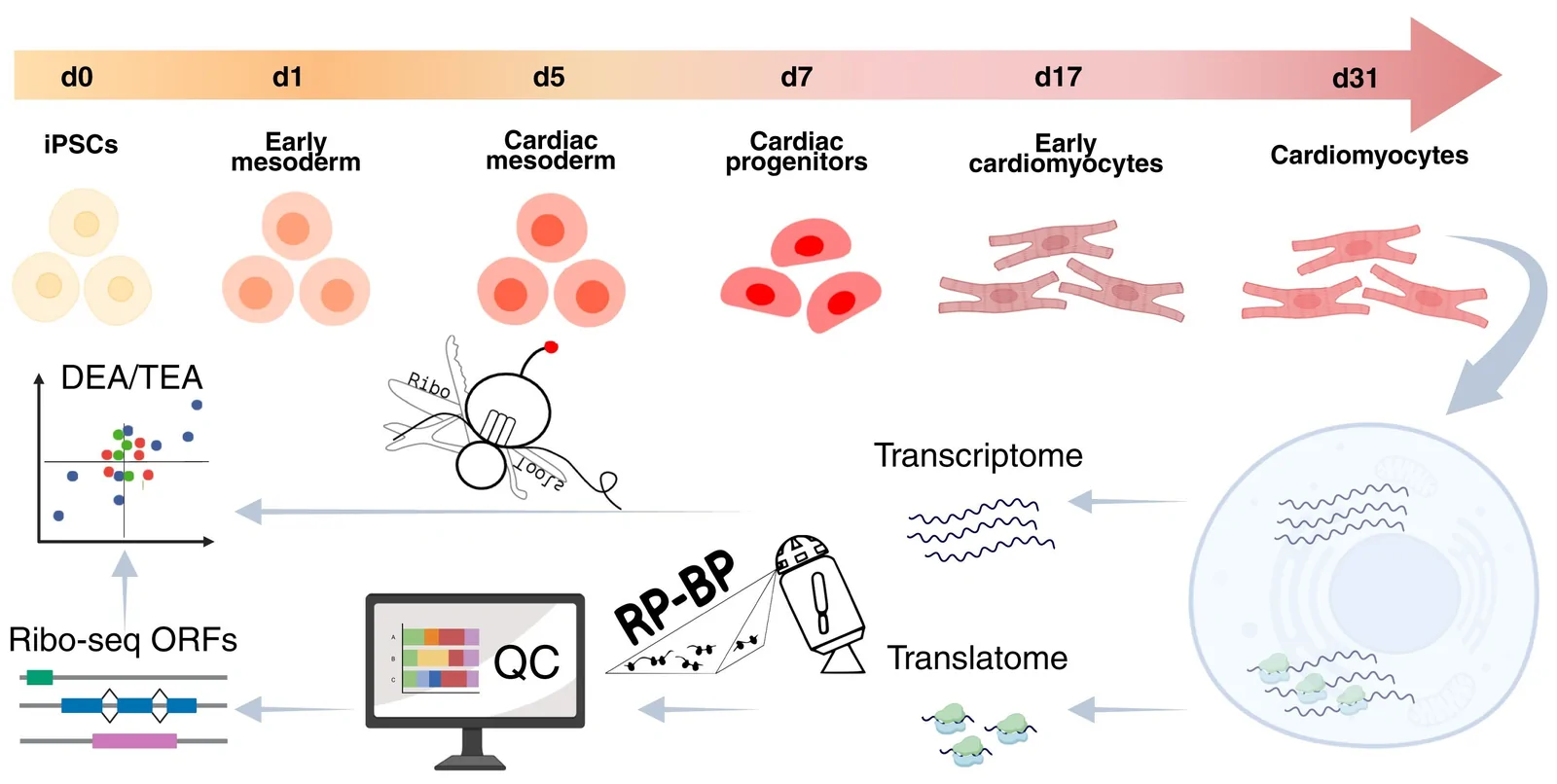

## Introduction

Ribosome profiling (Ribo-seq) is an RNA sequencing (RNA-seq)-based readout of RNA translation. Isolation and deep sequencing of ribosome-protected RNA fragments (RPFs, or ribosome footprints) provide a genome-wide snapshot of the translatome at sub-codon resolution. Since 2010, Ribo-seq has been widely applied to many organisms, tissues, or cells, revealing a dark translatome of thousands of unannotated open reading frames (ORFs) [1–3]. The identification, characterization, and annotation of small functional or regulatory ORFs continue to attract substantial interest [4, 5], together with efforts to understand how tissue-, stage-, or disease-specific translational regulation can be harnessed for both research and therapeutic applications [6–8]. Recent efforts have focused on developing consensus public resources, such as databases and web servers [9–13], and on defining standardized annotations and guidelines for non-canonical ORF research [14, 15]. Standalone, bundled, and online Ribo-seq-based tools have been developed for use as data resources, for ORF discovery and differential translation [16]. Most methods use the 3-nt periodic signature of translating ribosomes to identify Ribo-seq ORFs, including Rp-Bp [17]. Since its publication, Rp-Bp has been used in a number of studies where unsupervised selection of read lengths was required for robust periodicity estimation [18–22].

Although several methods have been developed specifically for translation efficiency analysis (TEA), the field currently lacks a robust, modular, end-to-end (E2E) solution that is actively maintained, well documented, and enables a principled classification of features into distinct layers of gene regulation. We present here Ribotools, a comprehensive toolbox that, in combination with Rp-Bp, provides an E2E solution for Ribo-seq quality control (QC), ORF discovery, and TEA. We provide a benchmarking pipeline for TEA tools, publicly available at https://github.com/eboileau/ribotools-benchmark, and compare Ribotools with available ORF discovery tools. We show that Ribotools stands out as the best all-around performer. We generated a time course dataset of differentiating human induced pluripotent stem cells (hiPSCs) into cardiomyocytes (CMs). Ribotools allowed us to reveal how translational regulation follows a highly dynamic trajectory, shifting from early buffering of lineage programs to translational forwarding during commitment, followed by suppression of signaling and remodeling pathways at later stages. Our results also include a comprehensive atlas of Ribo-seq ORFs, complemented by mass spectrometry, as well as interactive dashboards hosted at https://rpbp.dieterichlab.org/hIPSCCMdiff/profile_construction and https://rpbp.dieterichlab.org/hIPSCCMdiff/predictions. These data will serve as a resource and blueprint for investigating hiPSCs as a model of cardiac development.

## Materials and methods

### Maintenance of hiPSCs

The hiPSC line UMGi014-C clone 14 (isWT1.14) was provided by Lukas Cyganek (Stem Cell Unit, University Medical Center Göttingen). Cells were cultured in Stem MACS iPS Brew XF Medium (Miltenyi Biotec) under humidified normoxic conditions (37°C, 5% CO$_2$). The culture medium was changed daily. Cultures were routinely passaged at a ratio of 1:6 every 4–5 days following dissociation using Versene Solution (Thermo Fisher). Culture plates were coated with 17*μ*g/cm$^2$ Growth Factor Reduced Matrigel (Corning) prior to seeding. Cells were plated in Stem MACS iPS Brew XF Medium supplemented with 2 μM Thiazovivin (Millipore) for the first 24 h after dissociation.

### Differentiation of hiPSCs into ventricular cardiomyocytes

Human iPSCs were differentiated into ventricular CMs as previously described [23]. Briefly, cells were detached using Versene Solution (Thermo Fisher) and plated on 6-well culture plates pre-coated with 17 μg/cm^2^ Growth Factor Reduced Matrigel (Corning) using 150 000 cells/well in a final volume of 2 ml Stem MACS iPS Brew XF Medium supplemented with 2 μM Thiazovivin (Millipore) per well. Differentiation was started (day 0) when cells reached *> *95% confluency by replacing iPS culture medium with 3 ml/well Cardio Differentiation Medium [RPMI 1640 with GlutaMAX and HEPES (Thermo Fisher) supplemented with 0.2 mg/ml L-ascorbic acid 2-phosphate (Sigma–Aldrich), 0.5 mg/ml human recombinant Albumin (Sigma–Aldrich), and 4 μM CHIR99021 (Merck Millipore)]. The next day, an additional 3 ml Cardio Differentiation Medium were added on top, creating a CHIR99021 concentration gradient. On day 3, the medium was switched to Cardio Differentiation Medium supplemented with 5 μM IWP2 (Merck Millipore). Day 5 and onward, the medium was changed to 3 ml Cardio Differentiation Medium every 2 days. From day 7 onward, the medium was changed to 3 ml Cardio Culture Medium [RPMI 1640 with GlutaMAX and HEPES (Thermo Fisher) supplemented with 1× B27 (Thermo Fisher)] every 2 days. On day 10, metabolic selection was performed using Cardio Selection Medium [RPMI 1640 without glucose and HEPES, with 0.2mg/ml L-ascorbic acid 2-phosphate, 0.5 mg/ml human recombinant Albumin (Sigma–Aldrich), 4 mM lactate/HEPES] for 3 days. On day 15, cells were detached using 0.25% Trypsin ethylenediaminetetraacetic acid (Thermo Fisher) and replated in 2ml Cardio Culture Medium supplemented with 20% FCS (Thermo Fisher) and 2 μM Thiazovivin on Matrigel pre-coated 6-well culture plates. A second metabolic selection was performed on day 20 as described earlier. From day 23 onward, cultures were maintained in Cardio Culture Medium with regular media changes every 2–3 days.

### Sample collection

Samples were collected at days 0, 1, 5, 7, 17, and 31 post-differentiation start. For transcriptome and translatome analysis, cells were treated with cycloheximide (CHX) (100 μg/ml) for 5 min at 37°C, washed once with ice cold phosphate buffered saline (PBS)/CHX (100 μg/ml), and lysed in 600 μl of lysis buffer [20 mM Tris–HCl, pH 7.4, 5 mM MgCl$_2$, 150 mM NaCl, 1% Triton X-100, 100 μg/ml CHX, 1*×*cOmplete protease inhibitors (Roche)] per 6-well plate. One 6-well plate was used as one replicate. Lysates were clarified at 10 000 rpm for 10 min at 4°C, and the supernatants were snap-frozen in liquid nitrogen until all samples were collected. For mass spectrometry, cells were detached using a cell scraper into 1ml of ice-cold PBS. Cell pellets were collected by centrifugation (1200 rpm, 4°C, 10 min), washed once with ice-cold PBS, and snap frozen in liquid nitrogen.

### Ribosome profiling

Snap-frozen polysome lysates were thawed on ice. Ribosome footprinting was performed as previously described in Tuorto *et al*. [24]. Briefly, 400 μl of polysome lysates were digested with 8 μl RNase I (100 U/μl) and 4 μl TURBO DNase I (1 U/μl) for 45 min at RT with occasional shaking. To stop digestion 20 μl of RNasin ribonuclease inhibitor (40 U/μl, Promega) was added to each sample. Tubes were stored on ice, and 300 μl of each sample was loaded onto a 17.5%–50% linear sucrose gradient. Samples were centrifuged at 35 000 rpm for 2.5 h at 4°C in a Beckmann SW60 rotor. Gradients were eluted using a Foxy R1 Fraction Collector (Teledyne ISCO), and profiles were recorded by continuously monitoring absorbance at 254 nm. Fractions containing the monosome peaks in the recorded profiles were used for library preparation. Ribosome-protected footprint RNA was end-repaired with T4 polynucleotide kinase (Thermo Fisher) and size-selected for 28–31 nt on a 15% polyacrylamide TBE-UREA gel. Sequencing libraries were prepared using the NEBNEXT Small RNA Library Prep kit for Illumina (NEB). Libraries were pooled based on molarity and sequenced at GeneCore (EMBL) on a NextSeq 2000 platform.

### RNA sequencing

Total RNA was extracted from 100 μl of polysome lysate using TRIzol Reagent (Invitrogen) according to the manufacturer’s instructions. For each differentiation time point, two biological replicates were processed. Ribosomal RNA (rRNA) was depleted using the NEBNext rRNA Depletion Kit v2 (Human/Mouse/Rat) (New England Biolabs). Sequencing libraries were prepared from 200 ng total RNA using the NEBNext UltraExpress RNA Library Prep Kit (NEB #E3330L) according to the manufacturer’s instructions, with a fragmentation time of 15 min. Libraries were pooled based on molarity and sequenced at GeneCore (EMBL) on a NextSeq 2000 platform.

### Genome annotation

The human assembly GRCh38 was used with Ensembl human gene annotations (Ensembl release 102) to guide a *de novo* transcriptome assembly for the differentation of hiPSCs to CMs. Paired-end RNA-seq data were trimmed for residual adapter sequences and quality-filtered using Flexbar. Bowtie2 was used for *in silico* rRNA removal. The remaining reads were mapped using STAR. The transcripts were assembled using StringTie. The annotations were merged into a non-redundant set of features and compared with the reference annotation using GffCompare. All details and references are in the Supplementary Methods S1.

### Ribotools pipeline overview

The Ribotools pipeline supports multiple analysis workflows. It operates as a standalone solution to generate periodic (Ribo-seq) or trimmed (RNA-seq) alignments and read-count tables (quantification); it can be used for translation efficiency (TE/DE analyses) alone or in combination with Rp-Bp for streamlined QC (Fig. 1A). When used in conjunction with the abundance estimation pipeline, the TEA pipeline automatically generates the required input files. Alternatively, it can be run directly on count data produced by an external workflow. For analyzes based on Ribo-seq ORFs, Ribotools provides functionality to generate a dedicated GTF annotation, enabling the abundance estimation pipeline to quantify ORF-level expression and subsequently perform TE or differential expression analyses at the ORF level. The QC dashboard allows users to visualize read filtering, length distributions, periodicity estimates, metagene profiles, etc. Together with the ORF discovery pipeline, the corresponding dashboard provides multiple views to facilitate ORF discovery, including an integrated IGV browser for visual exploration of predicted Ribo-seq ORFs. The applications can be run locally on the user’s browser, remotely, or served to share results with collaborators. The Ribotools and Rp-Bp pipelines are designed to support the complete analysis workflow, from raw FASTQ files to the final set of translated Ribo-seq ORFs and/or translationally regulated features. Each pipeline can also be started from any intermediate step, provided the input data are appropriately prepared and organized (Fig. 1B). A single YAML configuration file can be used for all steps. The directory structure is automatically generated and used to identify existing data on the file system. In the background, Ribotools uses HTSeq for abundance estimation and DEseq2 to model TE. Differential TE is tested in a two-step procedure using a likelihood ratio test (LRT) within the DESeq2 negative-binomial GLM framework: LRT for the global model fit, and Wald tests with fold change threshold for extracting directional contrasts. Ribotools then automatically classifies significant features into four regulatory classes, according to the terminology defined in the deltaTE protocol [25]. All details and references are in the Supplementary Methods S2.

**Figure 1.**
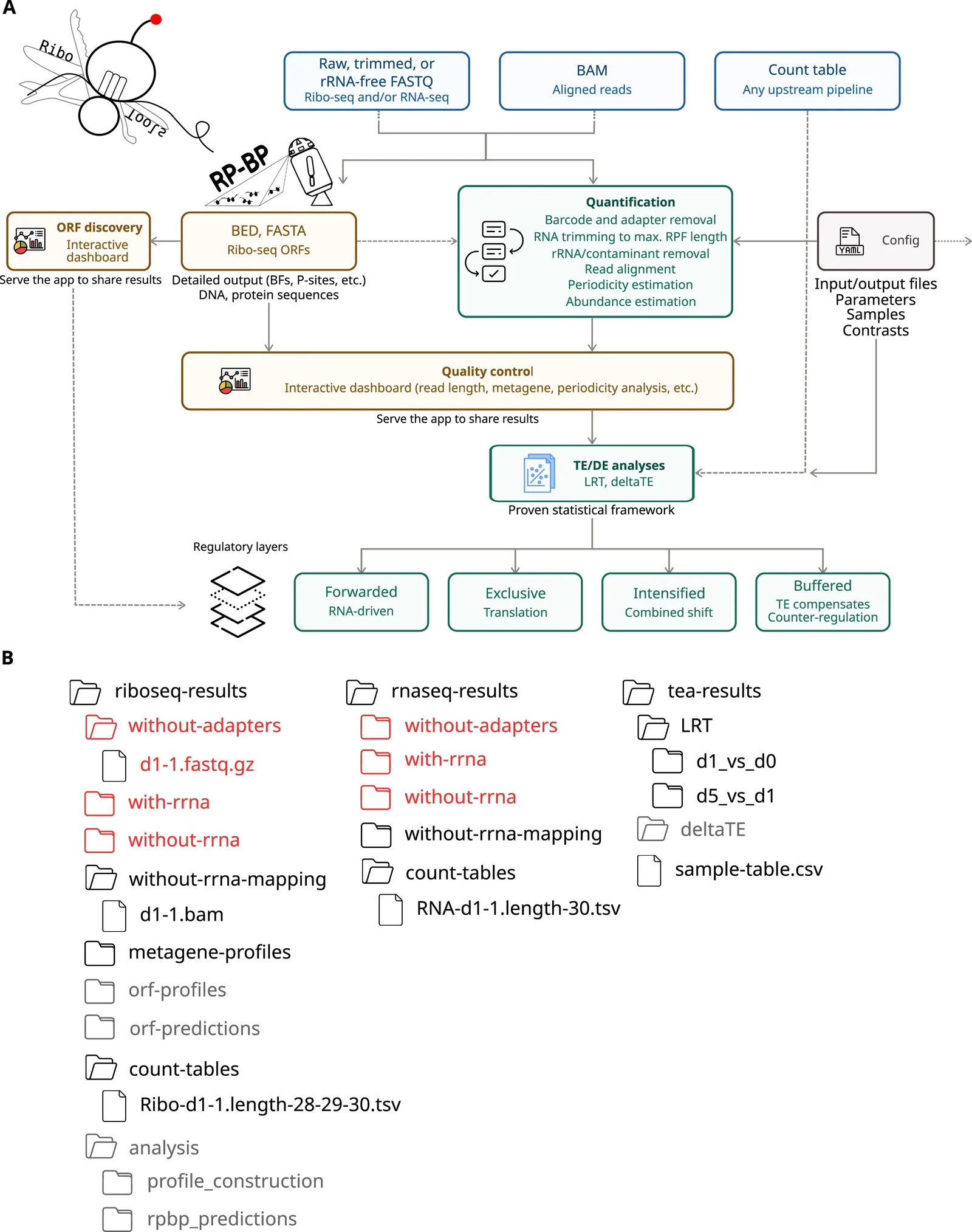
Overview of the Ribotools box. (**A**) Ribotools, together with Rp-Bp, supports periodicity estimation and Ribo-seq QC, ORF discovery, abundance estimation (using either annotated genes or Ribo-seq ORFs as features), and TEA. Ribo-seq abundance is quantified using only periodic RPFs, and reads from matched RNA-seq libraries are trimmed to the maximum periodic fragment length to minimize mapping bias. The E2E pipeline supports the complete analysis workflow but can also be started from any intermediate step, with the option to skip completed steps or overwrite existing results. A single YAML configuration file is used throughout the workflow. These steps can be configured via the configuration file or using command-line arguments. Two interactive dashboards facilitate QC and Ribo-seq ORF exploration and can be deployed to share results with collaborators. Finally, Ribotools is built on a robust statistical framework and automatically classifies features into distinct layers of gene regulation. (**B**) Output files directory tree. The first directory is given in the configuration file, and the remaining tree is automatically generated and used to subsequently identify existing files on the system. Directories in red can be deleted upon completion to save on disk space, or kept for QC. Directories in light gray are only present when performing QC, when running the ORF discovery pipeline, or when using the deltaTE approach (LRT is the default). Required input and expected output for each step are described in the online documentation. This image has been designed using resources from Flaticon.com.

### Rp-Bp pipeline overview

The pipeline consists of two modules: the ORF profile construction and the Ribo-seq ORFs prediction. The Bayesian approach was previously described [17]. In this new release, PyStan 2 was replaced with the CmdStanPy API. All models are now pre-compiled and shipped with the Bioconda package. Here, we used the newly generated transcriptome annotation to predict *de novo* translation events by directly assessing the periodicity of the Ribo-seq signal, without the need for model training or fine-tuning. Rp-Bp automatically assigns identifiers and labels to ORFs using their position relative to the annotated transcript-exon structure. The ORF identifiers include the host transcript and genomic coordinates, and the labels follow the standardized annotation of ORFs suggested by the TransCODE Consortium [14], enabling users and non-specialists to easily catalog alternative ORFs and recognize those that may be of particular interest, simplifying downstream analyzes. All details are in the Supplementary Methods S3.

## Results

### Ribotools stands out as the strongest all-around tool

Among tools specifically designed for TEA, Ribotools is the only fully modular pipeline, offering a one-step quantification together with a principled classification of regulated features into distinct layers of gene regulation (Supplementary Methods S4 and Supplementary Table S1). In a first benchmark, we evaluated the detection performance of Ribotools, Riborex [26], anota2seq [27], and the deltaTE method [25] (Supplementary Methods S4 – benchmark 1). Ribotools performed on par with deltaTE and marginally outperformed anota2seq in most test cases (Supplementary Results S1 and Supplementary Fig. S1). Riborex’s apparent advantage in translation-specific comparisons reflected its undifferentiated TE-interaction score rather than genuine superiority; it performed worst when all regulatory classes were considered, owing to its inability to detect abundance-regulated genes. Classification accuracy against the ground-truth labels was similarly high and consistent across all classifying tools for the abundance and background classes, moderate for translation and buffering, with anota2seq showing comparatively lower recovery of translation genes (Supplementary Results S1 and Supplementary Fig. S2A and B). In a second benchmark, we used a subset of our newly generated dataset and performed label permutation to quantify Ribotools’ empirical false positive rate (Supplementary Methods S4 – benchmark 2). This analysis yielded zero false positives at FDR *< * 0.05 under the null, supporting the specificity of the significant genes identified in the real data, despite the inherent limitation imposed by *n* = 2 replicates per condition (Supplementary Results S1 and Supplementary Fig. S2C). Finally, we performed a qualitative comparison of Ribotools with other available ORF discovery tools (Supplementary Results S2 and Supplementary Table S2). Among tools specifically designed for ORF calling and discovery, Ribotools/Rp-Bp is one of the few actively maintained options, with extensive online documentation and tutorials, comparable in this respect only to ORFik [28]. Ribotools and ORFik differ in functionality and implementation details, but only Ribotools/Rp-Bp provides interactive visualization dashboards. ORFik’s ORF translation prediction relies on a heuristic, supervised learning approach, whereas Rp-Bp builds on a probabilistic model designed for unbiased *de novo* discovery, providing full posterior distributions rather than point classifications [17]. In summary, Ribotools emerged as the only E2E, modular, and actively maintained TEA tool, consistently achieving the best overall performance in our benchmark, and providing, in combination with Rp-Bp, a robust workflow for Ribo-seq data analysis, QC, and ORF discovery.

### Transcriptome and translatome of hiPSC differentiation into cardiomyocytes

We used Ribotools to investigate how translational control evolves during cardiac lineage commitment *in vitro*. hiPSCs were differentiated into CMs and collected in duplicate at key developmental stages: hiPSCs (day 0), early mesoderm (day 1), cardiac mesoderm (day 5), cardiac progenitors (day 7), early CMs (day 17), and CMs (day 31). Each sample was subjected to transcriptome (RNA-seq) and translatome (Ribo-seq) profiling and analyzed with Ribotools.

#### Quality control

A demonstration of the QC dashboard for this dataset is hosted at https://rpbp.dieterichlab.org/hIPSCCMdiff/profile_construction. Each Ribo-seq library produced an average of 10.5 million mapped reads, of which ~6.7 million periodic reads were retained for subsequent analyzes (Fig. 2A and online). The Ribo-seq data showed high quality; reads were predominantly 28 or 29 long, and an average of 84% were mapped to the primary reading frame of the annotated coding sequences (CDSs) (Fig. 2B and online). Length distributions and metagene profiles are available for each sample to inspect read length periodicity, enabling users to visualize characteristic signatures of translation such as post-initiation pausing at the fifth codon position [29] (Supplementary Fig. S3A and online).

**Figure 2.**
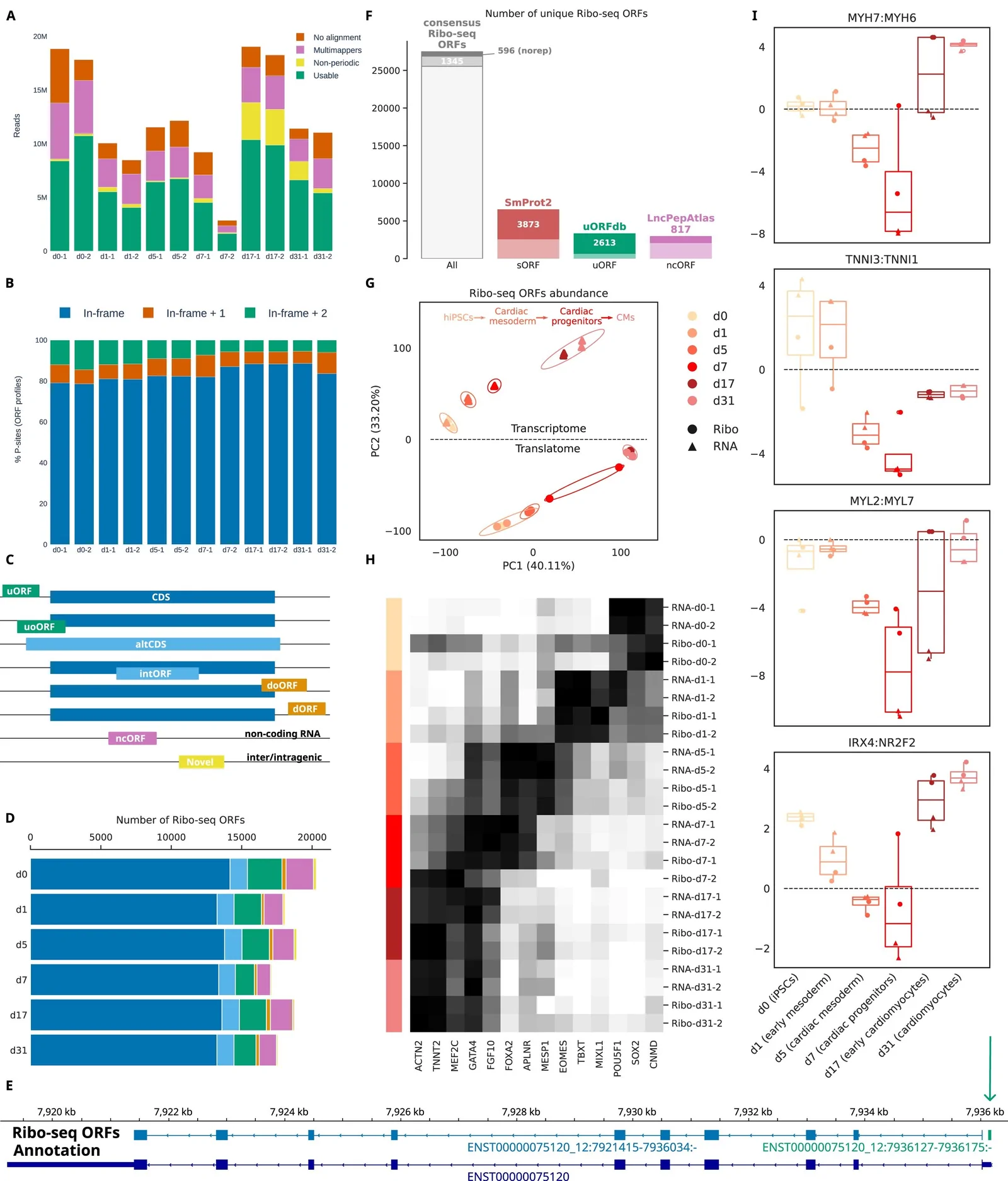
Transcriptome and translatome profiling capture cardiac lineage commitment and identifies unannotated Ribo-seq ORFs during hiPSC-differentiation. (**A**) Image taken from the QC dashboard. Stacked bars showing the number of reads classified as usable, non-periodic, multimapping, or not aligned, for each Ribo-seq library. Read filtering can also show the number of reads after *in-silico* rRNA removal for experimental protocol assessment. (**B**) Image taken from the QC dashboard showing the proportion of periodic RPFs assigned to each reading frame. (**C**) Image adapted from the ORF discovery dashboard. Schematic representation of the Ribo-seq ORF categories assigned according to their positions relative to annotated CDSs: upstream ORF (uORF), upstream overlapping ORF (uoORF), alternative CDS (altCDS), internal ORF (intORF), downstream overlapping ORF (doORF), downstream ORF (dORF), ORF in a transcript annotated as noncoding (ncORF), and novel intergenic or intragenic ORF (novel). (**D**) Bars with the total number of Ribo-seq ORFs predicted by Rp-Bp during hiPSC development to CMs, divided by developmental stage and ORF category. (**E**) Image adapted from the ORF discovery dashboard showing the integrated IGV browser and a predicted uORF upstream of the annotated SLC2A3 CDS. Rp-Bp assigned this ORF the identifier ENST00000075120 12:7936127–7936175:-. The ORFs are optionally assigned an extra identifier (c12riboseqorf16), matching that of the GENCODE consensus set of Ribo-seq ORFs, searchable via the ORF discovery dashboard. (**F**) Total number of Ribo-seq ORFs found during hiPSC development to CMs with evidence from the GENCODE consensus set of Ribo-seq ORFs (including non-replicated, or “norep” ORFs, found in only one experiment, i.e. not confirmed by any other independent research at the time), SmProt2 (for sORFs), uORFdb (for uORFs), and LncPepAtlas (for ncORFs). The numbers of top of each bar show how many Ribo-seq ORFs were found in each database, per category. Standardized ORFs were matched by coordinates; SmProt2, uORFdb, and LncPepAtlas were matched by amino acid sequence. (**G**) PCA of the RNA- and Ribo-seq abundances obtained with Ribotools for all Ribo-seq ORFs found in this study. Differentiation stages are colored from day 0 to day 31. Triangles represent RNA-seq and circles represent Ribo-seq samples. (**H**) Heatmap with scaled abundances for selected pluripotency, mesodermal, cardiac progenitor, and cardiac marker genes using variance-stabilized gene counts obtained with Ribotools. Differentiation stages are colored as in panel (G). (**I**) Box plots with MYH7:MYH6, TNNI3:TNNI1, MYL2:MYL7, and IRX4:NR2F2 ratios using variance-stabilized gene counts obtained with Ribotools across developmental stages (same legend as in panel G). Boxes indicate interquartile range; center line indicates median.

#### ORF discovery

The Ribo-seq ORF discovery dashboard, available at https://rpbp.dieterichlab.org/hIPSCCMdiff/predictions, showcases alternative translation events that occur during cardiac lineage commitment (Fig. 2C and online). As expected, Ribo-seq ORFs were found mainly within Ensembl-annotated CDSs, but an average of 1861 upstream ORFs (uORFs), 1425 ncORFs, and ~1218 variants of annotated CDSs were also translated at each developmental stage (Fig. 2D and online). Our *de novo* transcriptome allowed us to predict 392 completely novel, uncharacterized ORFs. A total of 1941 Ribo-seq ORFs were matched to the GENCODE consensus set of Ribo-seq ORFs (https://www.gencodegenes.org/pages/riboseq_orfs), of which ~95% are unannotated small ORFs (sORFs), mostly uORFs, such as a GATA4 uORF recently described [1, 30, 31], or c12riboseqorf16, upstream of the glycolysis gene SLC2A3, identified by Ribotools as translationally downregulated after day 17 (Fig. 2E and F, Supplementary Fig. S3B and C). Additional results can be found in the Supplementary Results S3 and Supplementary Fig. S4.

#### Downstream analyses using ORF counts

Principal component analysis (PCA) from Ribo-seq ORF counts, obtained with the Ribotools abundance estimation pipeline, delineated the progression from hIPSCs to CMs in a comparable developmental order at both transcriptome and translatome levels (Fig. 2G). Cells on day 0 and day 1 showed highly similar profiles, marked by the expression of pluripotency and early mesoderm markers (Fig. 2H). At days 5 and 7, cells progressed from cardiac-committed mesoderm to cardiac progenitors. From day 17 onward, they predominantly expressed CM markers. In general, the abundance profiles of cardiac-committed hiPSCs revealed a transition from low MYH7:MYH6, TNII3:TNNI1, MYL2:MYL7, and IRX4:NR2F2 on days 5 and 7, to higher ratios on days 17 and 31, confirming a shift from fetal to mature-like CMs (Fig. 2I).

In summary, we obtained a global snapshot of RNA translation across hiPSC differentiation into CMs, and the Ribotools box allowed us to demonstrate that hiPSC-CMs are a reasonable model to better understand cardiac lineage commitment.

### Translational control during hiPSC differentiation into cardiomyocytes

Using Ribotools, we were able to show how translational regulation follows a dynamic trajectory during CM differentiation, with multiple layers for commitment, execution, control, and stability (Fig. 3 and Supplementary Fig. S5). On day 1, coordinated changes mediated a wide spectrum of cellular responses crucial for cell differentiation, cell fate, and development in general (Fig. 3A). On day 5, cells experienced a major rewiring as they exited pluripotency and underwent lineage commitment. During early CM differentiation, genes involved in translation were predominantly translationally downregulated, consistent with coordinated repression of the translation machinery during the shift from proliferative to lineage-specific programs (Fig. 3B). These observations were supported by gene set enrichment analysis (GSEA), where we observed a negative enrichment for rRNA processing (NES −2.8), translation elongation (NES −2.6), translation initiation (NES −2.6), and cytoplasmic translation (NES -2.2), in which an important number of translationally downregulated ribosomal protein genes were part of the core enrichment set (Supplementary Methods S5 on functional enrichment analyses and Supplementary Table S3). These changes may reflect a shift in translational capacity and selectivity as cell acquire lineage-specific translational programs. Whether this transition involves altered ribosome composition or specialization remains to be determined [32, 33]. At the same time, key developmental cardiac pathways were transcriptionally activated, but translationally buffered (Fig. 3B and Supplementary Fig. S5A), suggesting a “timing and dosage” mechanism for fine-tuning cardiac protein expression, enabling a more precise temporal control than would be achievable through transcription alone. Compared to day 0, buffered pathways on day 5, as highlighted by GSEA, were enriched for the morphogenesis of cardiac septum (NES 2.3), cardiac muscle tissue (NES 2.2), and endocardial cushion (NES 2.1). These observations were partly confirmed by differential proteomics (Supplementary Methods S6 on protein correlation analysis and Supplementary Figs S6 and S7A–C), where upregulated proteins were still largely enriched for alternative lineage programs. By day 7, cardiac and myogenic genes transitioned from buffered to forwarded regulation, indicating lineage restriction and active execution of the differentiation program at the translational (Fig. 3C) and protein level (Supplementary Fig. S6 and Supplementary Fig. S7D and E). Persistent buffering of cardiac, lipid metabolic, and nuclear transport processes suggested ongoing fine-tuning and staged activation of functional and metabolic networks. As cells transitioned to maturation on day 17, they moved from a responsive/adaptive state to a more stable functional state, actively reducing remodeling and signaling plasticity, and shutting down alternative fate decision programs at the translational level (Fig. 3D). Although we observed refinement and reorganization at the translational level, changes in protein abundance remained predominantly associated with cardiac lineage and functional programs (Supplementary Fig. S6 and Supplementary Fig. S7E). On day 31, the system had reached a stable state in which protein output appeared to be actively maintained rather than dynamically remodeled (Supplementary Figs S5B, S6, and S7F). At this stage, and in the absence of changing conditions, translational regulation primarily serves to maintain protein homeostasis, with most pathways remaining stable through buffering mechanisms. Specifically, changes in RNA abundance were compensated by translation efficiency (i.e. a significant interaction term), resulting in stable RPF levels despite significant transcript-level changes (Supplementary Fig. S5B). Across most developmental stages, the differential proteomics were broadly concordant with the expectations of the Ribotools classification (Supplementary Methods S5, Supplementary Results S4, and Supplementary Fig. S8).

**Figure 3.**
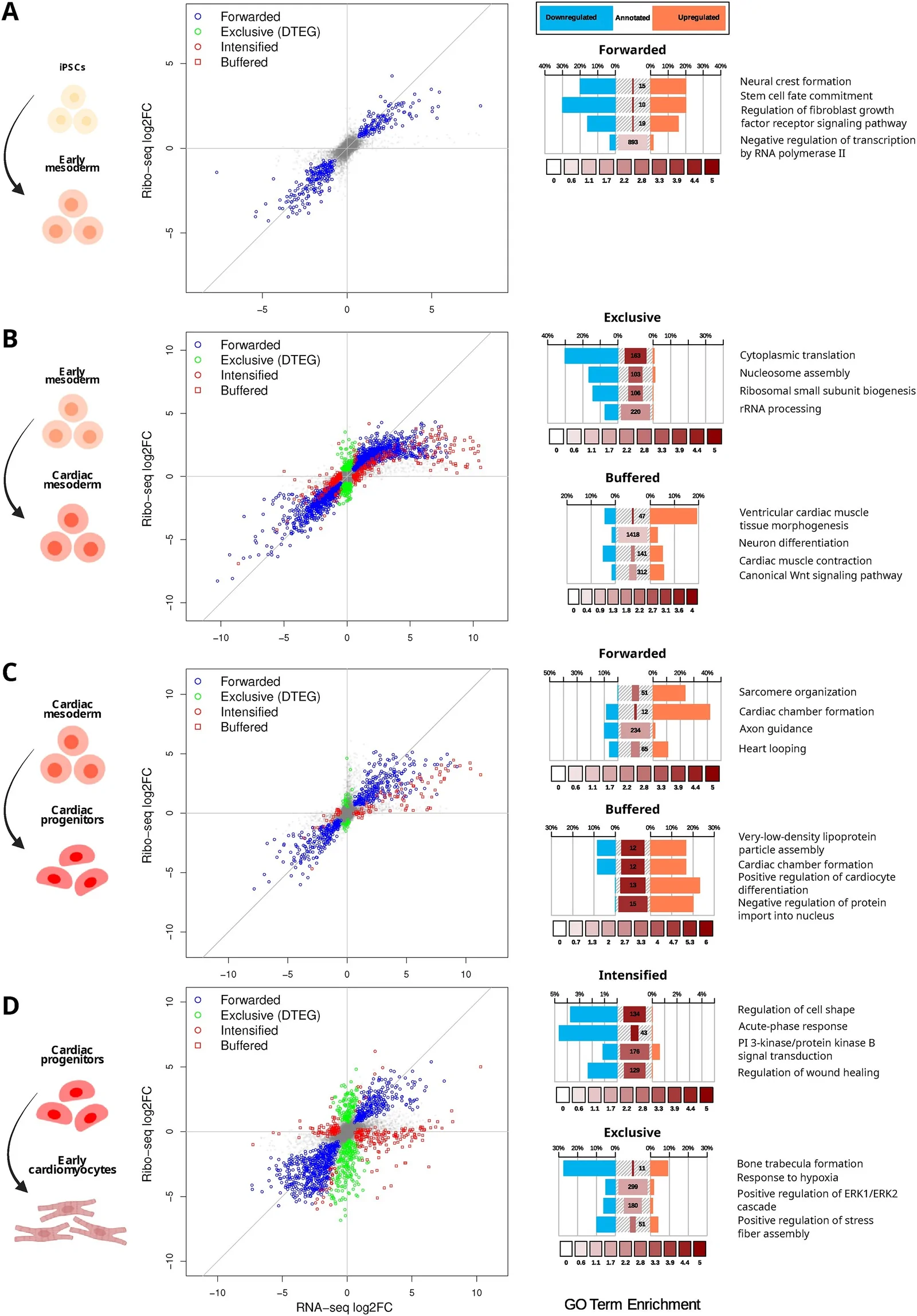
Transcription and translation are highly dynamic during differentiation. From top to bottom, the comparisons show (**A**) day 1 versus day 0 (from hiPSCs to early mesoderm), (**B**) day 5 versus day 1 (from early to cardiac mesoderm), (**C**) day 7 versus day 5 (from cardiac mesoderm to progenitor cells), and (**D**) day 17 versus day 7 (from cardiac progenitors to early CMs). Left: Schematic representation of the corresponding differentiation transition. Middle: Output from Ribotools. Scatter plots comparing RNA-seq log_2_FC (*x*-axis) to Ribo-seq log2FC (*y*-axis) for each gene. Genes were classified by Ribotools as forwarded, differential-translation exclusive (DTEG), intensified, or buffered. Right: Gene Ontology (Biological Process) over-representation analyses for selected classes, showing the proportion of differentially down- and up-regulated genes. The central column and associated color bar show the Gene Ontology term log enrichment score, together with the number of annotated genes. All details are in the Supplementary Methods S5. See also Supplementary Fig. S5.

## Discussion

The combined analysis of RNA- and Ribo-seq data is challenging and requires reliable tools. Here, we present the Ribotools box, an open-source, configuration-based, and modular package underpinned by Rp-Bp [17]. In contrast to naive ratio-based approaches [34, 35], changes in TE are inferred by testing the interaction term in a negative-binomial GLM fitted to RNA- and Ribo-seq counts [25, 26, 28]. Building on this established framework ensures methodological reliability while addressing practical gaps in the TEA landscape: dedicated QC tools focus on QC and visualization rather than complete analyses [36, 37], whereas TEA-only tools are designed for ready-to-use count data and lack Ribo-seq-specific QC; moreover, most are now deprecated or obsolete (refer to Supplementary Methods S4—Qualifying criteria—and Supplementary Table S1 for references). Ribotools is among the few well-documented and actively maintained E2E tools, providing QC, ORF discovery, and TEA “out-of-the-box” through an easy-to-use yet robust statistical framework. Like many widely adopted bioinformatics pipelines, its value lies not in introducing entirely new algorithms, but in providing a coherent and practical E2E solution.

Future work could include improved QC metrics and visualization, an interactive TEA dashboard, and support for specific protocols. Despite these limitations, Ribotools emerged as the strongest all-around performer, both in detection and classification and in the broader comparison of features across available TEA and ORF discovery tools (refer to Supplementary Results S2—Qualifying criteria and Ribotools stands out as the best all-around performer—and Supplementary Table S2 for additional references). Given the extensive evaluations of TEA methods in previous studies [25–27], our benchmark was not intended as another exhaustive comparison, but as a streamlined, pragmatic, and evolvable framework. The complete workflow is openly available and fully reproducible.

The active role of translation in cell-fate decisions during human cardiogenesis has long been recognized. More recently, Ribo-seq has been increasingly used to investigate cardiac reprogramming and to characterize the cardiac translatome at scale [1, 7, 8, 33]. Here, we demonstrate how Rp-Bp and Ribotools streamline the analysis of complex datasets and provide insights into the mechanisms underlying cardiac lineage commitment. We acknowledge that proteomics support for Ribo-seq ORFs remains limited, a well-recognized challenge, in particular for sORFs [14, 15]. Our objective is to demonstrate how Ribotools not only simplifies translatome profiling but also facilitates the early stages of ORF discovery. These extensive data represent a powerful resource to interrogate hiPSC-CMs as a model system, providing a source of potentially novel regulatory uORFs, alternative translation events, and much more.

## Supplementary Material

lqag130_Supplemental_File

## Data Availability

Raw sequencing data have been deposited in the NCBI Sequence Read Archive (SRA) under accession number PRJNA1447392. The mass spectrometry proteomics data have been deposited to the ProteomeXchange Consortium via the PRIDE partner repository with the dataset identifier PXD076601. Additional data supporting this study are available on Zenodo at 10.5281/zenodo.17899627. The Rp-Bp source code is available at https://github.com/dieterich-lab/rp-bp or https://zenodo.org/records/17974359. The Ribotools source code is available at https://github.com/eboileau/ribotools or https://zenodo.org/records/17974316. The source for the benchmarking pipeline is available at https://github.com/eboileau/ribotools-benchmark or https://zenodo.org/records/21409454. Packages and containers are available on Bioconda at https://bioconda.github.io/recipes/rpbp/README.html and https://bioconda.github.io/recipes/ribotools/README.html.
