# Supplementary material for: Rp-Bp and the Ribotools box for translatome profiling
Source: NAR Genom Bioinform. 2026 Nov 3;8(4):lqag130. doi: 10.1093/nargab/lqag130 (PMC13728661; doi:10.1093/nargab/lqag130)
Supplement: Supplementary file 1 [file lqag130_supplemental_file.pdf]

NAR Genomics and Bioinformatics, 2026, pp. 1–19

doi: https://doi.org/10.1093/nargab/
Application Notes

APPLICATION NOTES

Rp-Bp and the Ribotools box for translatome profiling: Supplementary data

Etienne Boileau,[1,2,3] Maja Bencun,[1,2,3] Francesca Tuorto,[4,5]
Isabel S. Naarmann-de Vries,[1,2,3] Pauline Fahjen,[6] Philipp Mertins[6,7]
and Christoph Dieterich[1,2,3,*]

[1]Department of Internal Medicine III (Cardiology, Angiology, and Pneumology), University Hospital Heidelberg, Im Neuenheimer Feld 669, 69120, Heidelberg, Germany, [2]Klaus Tschira Institute for Integrative Computational Cardiology, University of Heidelberg, Germany, [3]German Center for Cardiovascular Research (DZHK) – Partner site Heidelberg/Mannheim, Germany, [4]Division of Biochemistry, Mannheim Institute for Innate Immunoscience (MI3), Mannheim Cancer Center (MCC), Medical Faculty Mannheim, Heidelberg University, Franz-Volhard-Str. 6, 68167, Mannheim, Germany, [5]Center for Molecular Biology of Heidelberg University (ZMBH), DKFZ-ZMBH Alliance,Heidelberg, Germany, [6]Max Delbrück Center for Molecular Medicine (MDC),Robert-Rössle-Straße 10, 13125, Berlin,Germany and [7]Berlin Institute of Health at Charité – Universitätsmedizin Berlin,Charitéplatz 1, 10117, Berlin,Germany
[*]Corresponding author. christoph.dieterich@uni-heidelberg.de

Abstract

Supplementary information

Supplementary materials and methods

Methods S1. Genome annotation

A *de novo* assembled transcriptome annotation for human iPSCs differentiation into cardiomyocytes was generated using the RNA-seq data. Paired-end RNA-seq reads were first trimmed and filtered using Flexbar [12] with the following options 'flexbar -n 15 -z GZ -m 30 -u 0 -q TAIL -qt 28 -a adapters.fa -qf sanger -j'. Common contaminants were removed with Bowtie2 [19] using a built-in index combining mitochondrial and rRNA sequences with 'bowtie2 --no-unal --omit-sec-seq --threads 20 --mm --seed 1337 --time --un-conc-gz'. Remaining reads were mapped using STAR [11] with 'STAR --runThreadN 40 --genomeLoad NoSharedMemory --readFilesCommand zcat --outReadsUnmapped Fastx --outSAMattributes NH HI AS nM NM MD jM jI XS --outSJfilterOverhangMin 15 15 15 15 --outFilterMultimapNmax 20 --chimMultimapNmax 20 --outFilterScoreMin 1 --outFilterMatchNminOverLread 0.7 --outFilterMismatchNmax 999 --outFilterMismatchNoverLmax 0.05 --alignIntronMin 20 --alignIntronMax 1000000 --alignMatesGapMax 1000000 --alignSJoverhangMin 15 --alignSJDBoverhangMin 10 --alignSoftClipAtReferenceEnds No --chimSegmentMin 15 --chimScoreMin 15 --chimScoreSeparation 10 --chimJunctionOverhangMin 15 --sjdbGTFfile GRCh38.102.gtf --quantMode GeneCounts --twopassMode Basic --chimOutType Junctions'. Soft clipping was removed and the output SAM file was passed to samtools [9] for compression, sorting and indexing. To reconstruct *de novo* transcriptomes for each sample, guided by a reference transcriptome annotation (Ensembl release 102), Stringtie [31] was used 'stringtie -G GRCh38.102.gtf -p 2 -v --rf --conservative', followed by 'stringtie --merge -G GRCh38.102.gtf -p 2 -v'. Finally, the annotations were merged into a non-redundant set of features and compared with the reference annotation using Gffcompare [30] 'gffcompare -r GRCh38.102.gtf -V -R'. The resulting GTF annotation is publicly available on Zenodo [3].

Methods S2. Ribotools pipeline overview

The Ribotools pipeline comprises four main steps: *(i)* preparing a configuration file, *(ii)* preparing the annotation (when running the abundance estimation pipeline), *(iii)* running the abundance estimation pipeline, and *(iv)* running the TEA pipeline. For analyses based on Ribo-seq ORFs, the Rp-Bp pipeline has to be run beforehand, and its output is used directly as input to Ribotools for abundance estimation and TE analysis. These steps are described in detail in the online documentation, which also provides additional examples at https://ribotools.readthedocs.io.

Abundance estimation

Ribo-seq abundance is estimated from ribosome-protected fragments (RPFs) using only reads exhibiting the characteristic 3-nt periodicity, estimated using the automatic Bayesian Periodic fragment length and ribosome P-site offset Selection (BPPS) [27]. To minimize mapping bias, matched RNA-seq reads are trimmed to the maximum periodic fragment length of their corresponding Ribo-seq sample before abundance estimation. Trimming and quality filtering is performed using Flexbar [12], *in silico* rRNA removal using Bowtie2 [19], read alignment using STAR [11], and abundance estimation using HTSeq [32]. Parameters and options can be specified either in the configuration file or via command-line arguments.

© The Author 2026. Published by Oxford University Press. All rights reserved. For permissions, please e-mail: journals.permissions@oup.com

Differential translation

LRT method

Differential translational efficiency is tested in a two-step procedure with a likelihood ratio test (LRT) using DESeq2: LRT for the global model fit, and Wald tests with 'lfcThreshold' for extracting directional contrasts [26]. All RNA- and Ribo-seq counts are jointly modeled with the full design $assay + condition + assay : condition$, where $assay$ is a factor distinguishing RPF from RNA-seq libraries and $condition$ distinguishes treatment, or condition, from control, or reference. The test statistic is obtained by comparing this full model with the reduced model $assay + condition$. The LRT p-value therefore reflects the significance of the interaction term, *i.e.* the condition effect that differs between assays, without being tied to the direction or magnitude of the fold change. The fold changes are the maximum likelihood estimates (MLE), not a shrunken value, and represent how much the RPF/RNA ratio changes between conditions on the log2 scale. No fold change threshold can be set on the LRT interaction p-value. Separately, standard Wald tests with a fold change threshold are used to test the main effect of condition on RNA counts alone and on Ribo-seq counts, expressed as a linear combination of coefficients. Shrunken log2 fold changes for RNA and RPF are computed using empirical Bayes [36]. P-value adjustment uses Independent Hypothesis Weighting (IHW).

deltaTE method

This method re-implements the approach of Chothani *et al.* [5] with several improvements. Three separate DESeq2 models are fitted: (1) An interaction (full) model is fitted to all samples jointly. A Wald test is applied to the last coefficient with an fold change threshold to test whether the condition effect on Ribo-seq counts significantly exceeds the condition effect on RNA counts, *i.e.* a non-zero change in TE. IHW adjustment is applied as above. The interaction fold change is not shrunken. (2) A Ribo-seq marginal model is fitted separately with design $condition$. A Wald test with fold change threshold tests the condition effect on Ribo-seq. Fold changes are shrunken using either apeglm [49] or ashr [36], depending on the chosen option. (3) An RNA-seq marginal model is fitted separately with design $condition$ using the same procedure as above. The key differences from the original deltaTE protocol are: the original deltaTE extracts the interaction coefficient from a model fitted to all samples simultaneously, whereas Ribotools subsets to the relevant contrast before fitting; Ribotools applies an explicit fold change threshold to all Wald tests, which the original deltaTE code does not; and Ribotools applies IHW for p-value adjustment. In addition, parameters such as 'lfcThreshold' or FDR thresholds as well as fold change shrinkage method can be passed as argument. Multiple contrasts can be run at once, without the need to prepare separate count matrices and/or metadata tables. Batch effects can optionally be included.

Regulatory classes

Ribotools automatically classifies features into four classes, according to the terminology defined in the deltaTE protocol [5]. The definition of these classes is summarized below. Translationally *forwarded* features have a significant change in RNA and RPF at the same rate, without a change in TE ($padj.dte > \alpha$, $padj.ribo < \alpha$, $padj.rna < \alpha$). *Exclusive* features are differentially translated features driven by a condition effect that is significant for RPF ($padj.dte < \alpha$, $padj.ribo < \alpha$, $padj.rna > \alpha$). *Intensified* and *buffered* features take into account the direction of fold change. Translationally *intensified* features have a significant change in TE that acts with the effect of transcription (all three significant, $log2FC.dte \times log2FC.rna > 0$, *i.e.* sign($\Delta TE$) = sign(RNA log2FC)). Translationally *buffered* features have a significant change in TE, but not in RPF, that counteracts the change in RNA ($padj.dte < \alpha$, $padj.ribo > \alpha$, $padj.rna < \alpha$) or are under active counter-regulation (all three significant, $log2FC.dte \times log2FC.rna < 0$, *i.e.* sign($\Delta TE$) $\neq$ sign(RNA log2FC)).

Commands used

For the main results, Ribotools v2.0.0 was installed from Bioconda [15] with 'conda create -n ribotools ribotools'. The programs were run on a server running Debian GNU/Linux 12 (Linux kernel 6.1.174-1, x86_64) using Slurm 22.05.8. Unless otherwise stated, default options and parameters were used, as described in the online documentation. Indices and annotation were prepared with Rp-Bp (Supplementary Methods S3) The abundance estimation pipeline was run for gene features with 'run-htseq-workflow ribo config.yaml --run-all --trim-rna-to-max-fragment-size --ribo-config config.yaml --htseq-options "--stranded yes" --stranded reverse --rna-config config.yaml --mem 250G --keep-intermediate-files'. The sample table was prepared with 'get-sample-table config.yaml' and the TEA pipeline with 'run-tea config.yaml'. To estimate TE using Ribo-seq ORFs, a GTF file was first created using the predicted Ribo-seq ORFs with 'get-gtf-from-predictions hiPSC-CMs.Ribo-seq-ORFs.bed.gz hiPSC-CMs.Ribo-seq-ORFs.gtf --num-cpus 6'. The abundance estimation pipeline was run for Ribo-seq ORFs, as described above, but with the following additional arguments '--htseq-options "--idattr orf_id" "--additional-attr orf_type" "--additional-attr gene_name" "--stranded yes" --gtf hiPSC-CMs.Ribo-seq-ORFs.gtf'. In each case, calls to the abundance estimation pipeline were submitted to Slurm using '--use-slurm --num-cpus 6'. The sample table was prepared with 'get-sample-table config.yaml' and the TEA pipeline with 'run-tea –orfCol 2 –symbolCol 3 config.yaml'. The resulting count tables and TE results (for genes and Ribo-seq ORFs) are publicly available on Zenodo [3].

Methods S3. Rp-Bp pipeline overview

Ribo-seq ORFs were predicted with Rp-Bp (included in the Ribotools distribution). Briefly, when aligned by their P-site positions, footprints from actively translating ribosomes exhibit a characteristic 3-nt periodicity. Rp-Bp exploits this periodic signal through its automatic Bayesian Periodic fragment length and ribosome P-site offset Selection (BPPS). It then applies Bayesian model selection and inference, using Markov chain Monte Carlo to estimate the posterior probability that each ORF is actively translated [27]. The Rp-Bp pipeline consists of three steps: *(i)* preparing a configuration file, *(ii)* preparing the annotation (performed once for each reference genome), and *(iii)* running the ORF discovery pipeline. These steps are described in detail in the online documentation, which also provides additional examples at https://rp-bp.readthedocs.io.

Commands used

The following command was used to prepare the annotation 'prepare-rpbp-genome config.yaml –mem 400G'. To run the ORF discovery pipeline, we used 'run-all-rpbp-instances config.yaml --mem 250G --keep-intermediate-files --merge-replicates --run-replicates'. For quality control, we used 'summarize-rpbp-profile-construction config.yaml'. In each

case, calls were submitted to Slurm using '--use-slurm --num-cpus 6'. Finally, to summarize predictions, we used 'summarize-rpbp-predictions config.yaml --no-replicates --min-samples 2 --match-standardized-orfs'. The predicted Ribo-seq ORFs in BED and FASTA (protein sequences) are publicly available on Zenodo [3].

Methods S4. Benchmarking of TEA tools

The benchmarking workflow was implemented as a Snakemake pipeline [18]. Each rule was executed in a dedicated environment to isolate dependencies per tool and ensure full reproducibility. The pipeline code, the published count matrices, the environment specifications, the configuration files, and additional practical information are publicly available at https://github.com/eboileau/ribotools-benchmark. All benchmarks were performed on a Debian GNU/Linux 12 (Linux kernel 6.1.174-1, x86_64) machine, equipped with two Intel Xeon E5-2650 v3 processors (40 hardware threads) and 251 GB of RAM. Snakemake scheduling hints were set globally in a default profile and, in addition, processes memory and CPU usage were capped at the OS level.

Two benchmarks were defined: benchmark 1 assessed the detection and classification performance of each tool against the simulated data from Oertlin *et al.* [28], with known ground truth; while benchmark 2 quantified the empirical false positive rate of each tool on a real biological dataset through label permutations.

Benchmark 1

We used the simulated data from Oertlin *et al.* (Supplementary File 1) [28], and evaluated each tool's output against the provided ground-truth, composed of four exclusive classes: translation (exclusive translational regulation), buffering (mRNA-level change compensated at the translational level), abundance (congruent change in mRNA and ribosome occupancy), and background (no regulation). All tools were evaluated against the full set of genes present in the input count matrices. Four test cases were defined based on the ground-truth classes: *(1)* Translation strict (TP = translation; TN = background, abundance, and buffering combined), *(2)* Translation only (TP = translation; TN = background only, with abundance and buffering excluded to remove class ambiguity), *(3)* Any regulated (TP = translation, abundance, or buffering combined; TN = background), and *(4)* Buffering (TP = buffering; TN = background, translation, and abundance combined). The detection performance was assessed via receiver operating characteristic (ROC) analysis using the tool's continuous score (-log10 adjusted p-value) as the ranking statistic. This score is class-dependent for classifying tools, but the analysis itself was independent of any class label output. This test evaluates how well a tool's ranking score separates a defined positive set from a defined negative set, based on the ground truth labels. The area under the ROC curve (AUC) was computed for each tool and test case, with 95% confidence intervals (DeLong's method).

For classifying tools that output an explicit classification of genes into regulatory layers, classification accuracy was evaluated separately using per-class precision, recall, F1 score, and Matthews correlation coefficient (MCC). The narrower anota2seq class labels were used, with 'translation' for *exclusive*, 'abundance' for *forwarded*, and 'buffering' for *buffered* (see Supplementary Methods S2 for regulatory layers classification). The *intensified* class was considered a sub-case of the mRNA abundance class, where ΔTE is non-zero and amplifies the RNA change. Features classified as *intensified* by Ribotools and deltaTE were thus re-labeled as 'abundance'. All analyses were performed in R using the pROC package [35] for ROC/AUC estimation and ggplot2 [44] for visualization.

Qualifying criteria

Computational TEA tools were considered for inclusion in this benchmark if they met the following criteria. First, they had to show evidence of active or recent maintenance, defined as at least one substantial code commit or release within approximately the past five years; tools with no commit history or documented updates beyond this window were excluded as deprecated. Second, they had to be installable and run in a standard computing environment with at least R > 4.0 for R-based tools, and Python > 3.0 for Python-based tools; otherwise they were considered obsolete. Tools that were deprecated but could still be installed and run successfully under these minimum version requirements were retained, provided they met all other inclusion criteria. Third, they had to accept raw or minimally pre-processed Ribo- and RNA-seq count matrices as direct input. Fourth, they had to provide sufficient documentation to allow for correct usage and interpretation of their output. Tools that did not meet any of these four criteria were excluded from the benchmark, namely Babel (deprecated) [29] and Ribodiff (obsolete) [48]. Xtail [46] was installed, but did not run – not even on their own example data – and was therefore excluded. Except for a minor update in 2025, this package is considered deprecated. Three additional packages were excluded, namely tRanslatome [38], XPRESSyourself [2], and ORFik [40]. We were unable to find an author-maintained GitHub repository or equivalent for tRanslatome, except that from the Bioconductor's infrastructure, which showed no recent commits. We estimated that the last real code change was approximately 2020 or earlier. XPRESSpipe shows recent commit activity, but not XPRESSplot. The resolution of Conda dependencies is documented as problematic. Moreover, it did not satisfy the third criterion; we were unable to find reliable documentation regarding whether the package can handle arbitrary count matrices. The TE approach in both tRanslatome and XPRESSyourself is a simple ratio method and, therefore, of little interest, considering prior published benchmarks. ORFik was excluded because its main TEA function requires an ORFik experiment object, which wraps paths to the file system to build its own count tables, and therefore does not accept arbitrary matrices. Our initial attempt to bypass this requirement failed. ORFik is an E2E tool currently under active development, but its documentation is overwhelming and its usage demands entry-level to intermediate R scripting skills. Internally, it re-implements the deltaTE protocol, but its classification is less granular for the mRNA abundance case, and it introduces no new categories. Finally, we also considered tools such as RiboDiPA [21], diricore [25], RiboTools [20], RiboGalaxy [13], RiboToolkit [24], and RiboWave [47], which have been previously characterized as 'translation analysis tools'. RiboDiPA, diricore, and RiboTools are not specifically TE tools, *e.g* RiboDiPA performs differential pattern testing on P-site counts, diricore estimates amino-acid-specific ribosome stalling/dwell-time signal, and RiboTools, a Galaxy-based tool, detects stop codon read-through events and codon occupancy. Except for RiboDiPA, we found no evidence of recent updates or no reliable source code repository, and no standalone

installation instructions for the other two. RiboGalaxy and RiboToolkit are both online/web-platform tools. RiboGalaxy is a Galaxy-based browser platform that provides a suite of tools tailored for Ribo-seq data processing and visualization. RiboToolkit offers functionality within a web browser similar to that offered by RiboGalaxy. While RiboToolkit includes TEA, RiboGalaxy does not. However, RiboToolKit only accepts uploaded count tables through its web interface with no standalone package. RiboWave provides TE estimation as a secondary feature, but its commit history is over eight years old, and it does not provide a standalone installable package.

As an exception to these criteria, we included the deltaTE method [5]. Although deltaTE is provided as a published protocol rather than a package (it cannot be installed), and although it has no official documentation and its source repository is not maintained, we included it as a comparison to the implementation of Ribotools, which introduces some minor variants (see Supplementary Methods S2). To that effect, the script was taken from GitHub on the master branch, commit 60b61ca (Apr. 2021), patched to handle different conditions, and included in the Snakemake pipeline. This thus leaves only four different tools for this benchmark: anota2seq, Riborex, deltaTE, and Ribotools (LRT and deltaTE). See Table S1 for a summary.

Benchmark 2

To assess the false positive rate of each tool under null conditions, we performed a label permutation analysis using a subset of the data presented in this study, specifically samples from days 1 and 5. All valid permutations of labels were enumerated and applied simultaneously to the Ribo- and RNA-seq count matrices, preserving the pairing between assays (two replicates per day, resulting in four genuinely distinct non-original permutations). For tools implementing separate statistical tests for RPF, RNA abundance, and translational efficiency (TE interaction term), the empirical false positive rate was computed independently for each test as the proportion of genes reaching FDR < 0.05 among all tested genes. The proportion of true null hypotheses were estimated using the qvalue package [16].

Qualifying criteria

Computational TEA tools were considered for inclusion in this benchmark if they met the criteria defined for benchmark 1 (Supplementary Methods S4 – benchmark 1). Additional requirements were the ability to handle datasets with as few as two replicates per condition and to accept a user-defined FDR threshold as a parameter. These constraints left, for all practical purposes, only Ribotools as a qualifying tool. Since the statistical approach implemented in Ribotools has been shown to perform comparably to the deltaTE method, and since the deltaTE Wald-based and LRT-based implementations were also compared, only the LRT implementation was included here.

Methods S5. Downstream analyses

ORF discovery

RNAcode [43] was used to predict protein-coding regions from multiple sequence alignments. The software was installed using ‘mamba install -c bioconda bx-python rnacode‘. The Multiz30way multiple sequence alignment dataset, comprising 30 vertebrate species, was downloaded from the UCSC Genome Browser. Ribo-seq ORFs in BED12 format were converted to MAF format, and RNAcode was run on each ORF using default parameters. For each ORF, the highest in-frame RNAcode score was retained.

PhyloCSF [23] was used to assess the protein-coding potential of the predicted ORFs. Sequences with a deciban (log-odds) score greater than 0 were considered likely to be protein coding. PhyloCSF was installed using ‘mamba install -c bioconda phylocsfpp‘. Ribo-seq ORFs in GTF format were converted to GFF using ‘gffread -T -L‘. Precomputed PhyloCSF tracks for the hg38 genome assembly were downloaded from the Broad Institute and used to score ORFs with the ‘annotate-with-tracks‘ option. For each ORF, the highest in-frame PhyloCSF score was reported.

InterProScan was used to annotate predicted protein sequences. InterProScan 5 was installed locally according to the official documentation and run on the translated Ribo-seq ORF protein sequences in FASTA format using the options ‘–goterms –pathways -f TSV‘, enabling the assignment of protein domains, Gene Ontology (GO) terms, and pathway annotations.

Functional enrichment analyses

For each developmental stage, over-representation analysis (ORA) was performed with topGO [1], separately for each regulatory layer identified by Ribotools. Effect sizes (log2 fold changes) and adjusted p-values were defined according to the regulatory class: RPF for *intensified* and *exclusive* features, RNA for *buffered* features, and the average of fold changes together with Fisher's method for combining p-values for *forwarded* features. The background universe consisted of all expressed genes (17,902 genes). Results were visualized using the R package CellPlot. Gene set enrichment analysis (GSEA) was performed using the ‘GSEA’ function from the R package clusterProfiler [45].

Methods S6. Proteomics

LC-MS proteomics

The cell pellets were lysed in SDC Buffer (1% sodium deoxycholate, 150mM NaCl, 100mM Tris, 1mM EDTA) at 95°C for 10 minutes, and protein concentration was determined using the Pierce Protein-BCA assay kit (Thermo Fisher Scientific). Protein disulfide bonds were reduced with 10mM dithiothreitol, and free thiol groups were alkylated in 40mM chloroacetamide. Nucleic acids were removed by incubation with 25 U Benzonase. 100μg of protein were digested overnight at 37°C using Trypsin/LysC at a 1:50 (enzyme:protein) ratio, and 30μg of protein were digested using AspN at a 1:200 ratio. Digestion was stopped by addition of formic acid to a final concentration of 1%, and the samples were desalted using the StageTip approach [34]. 500ng of peptides per sample were injected onto an Orbitrap Astral MS coupled to a Vanquish Neo LC system (Thermo Fisher Scientific). Peptides were separated on an in-house packed column (20cm */times* 75μm inner diameter) containing 1.9μm C18 ReproSil particles at a flow rate of 350nL/min. A gradient was applied using solvent A (3% acetonitrile, 0.1% formic acid) and solvent B (90% acetonitrile, 0.1% formic acid). Solvent B was increased from 2% to 7% in 1 minute, to 20% over 9 minutes, to 30% over 7.5 minutes, and to 60% over an additional 2 minutes. In data-independent acquisition mode, full MS scans were acquired at 240,000 resolution with a normalized AGC target of 500%, a mass range of 380-1100 m/z, and a maximum injection time of 3ms. Each full scan was followed by MS2 scans using isolation windows of 2 m/z across a precursor

m/z range of 380-980 m/z, with a normalized AGC target of 500%, a normalized HCD collision energy of 25%, and a maximum injection time of 3 ms. Raw data were analyzed using directDIA in Spectronaut version 20.3, separately for each endopeptidase. Default settings were adjusted by adding deamidation (NQ) as a variable modification and adjusting the run-wise protein q-value cutoff to 0.01. For AspN-digested samples, cleavage was specified as N-terminal to aspartic acid. Spectra were searched against a human UniProt database (downloaded 04/2025) supplemented with the predicted protein sequences.

Differential proteomics analysis

Quantitative data at the peptide-level were processed using the DEP2 workflow (https://mildpiggy.github.io/DEP2/-index.html). Missing values were imputed separately within each experimental condition using a hybrid strategy: peptides with at least one observed replicate were imputed by BPCA, whereas peptides missing across all replicates of a condition were imputed using the left-censored MinProb method. This condition-specific approach was used to preserve stage-dependent abundance patterns and to avoid cross-condition signal leakage during imputation. The peptides were then normalized using median differences before aggregating to proteins for differential testing. The resulting proteins were tested at a significance threshold of 0.01, log2 fold change threshold of 2, and FDR were reported after Benjamini-Hochberg correction.

Protein correlation analysis

For each developmental stage, Spearman correlation was computed between protein log2 fold-change and the corresponding RNA or Ribo-seq log2 fold-change, as reported by Ribotools, separately for each regulatory layer. Only genes assigned to each class by the TEA analysis were used; no filtering on protein-level significance was applied.

Supplementary results

Results S1. Benchmarking of TEA tools

Benchmark 1

Oertlin *et al.* reported superior performance of anota2seq in detecting differential translation compared to Babel and Xtail, while Chotani *et al.*, in a subsequent evaluation, showed that deltaTE outperformed anota2seq, RiboDiff, and Riborex, particularly in the presence of batch effects [28, 5]. Here, we used the simulated dataset from Oertlin *et al.*, which provides ground truth class labels for translation, buffering, mRNA abundance, and background, to benchmark Ribotools, anota2seq, Riborex, and the deltaTE method. Since Ribotools builds on DESeq2 [26], a robust and well-established statistical framework for count-based differential expression analysis, and given that previous benchmarks have already provided extensive comparisons, our primary aim was to provide a lightweight, extensible, and reproducible benchmarking pipeline rather than an exhaustive evaluation of each tool's capabilities. While Chotani et al. reported dramatic differences in runtime across tools on larger datasets [5], such differences are compressed on the small dataset used here: Ribotools, deltaTE, and Riborex ran in seconds; anota2seq took ∼4 minutes. All tools used less than 1GB of memory. Wall-clock time, peak memory usage, and other resource usage are part of the benchmarking pipeline's standard output.

The detection performance, assessed independently of any class assignment and using each tool's ranking score, was broadly comparable in most test cases (Figure S1). All classifying tools derive their ranking score from a class-specific score. Riborex, the only non-classifying tool, uses a single undifferentiated score for all tests, built on DESeq2-style interaction model. This is not surprising if Riborex achieved the highest AUC in Translation (strict) and Buffering, where the positive class is largely defined by a TE interaction signal. In Any regulated, where abundance genes are included among the true positives, Riborex remained blind to the abundance-regulated subset of the positive set; classifying tools can detect these genes independently of any TE effect. To isolate raw detection power, we restricted the true negative set to background only in Translation (only), removing class ambiguity. The resulting convergence of all tools to comparably high AUC confirmed that Riborex's earlier advantage only reflected its direct targeting of the TE interaction signal rather than any general superiority in detecting translation. Anota2seq consistently ranked as the lowest-performing among classifying tools across all test cases, except Buffering. By correcting for RNA-RPF co-variation, it may assign lower scores to translation genes showing partial RNA co-regulation, a pattern that Ribotools and deltaTE can capture through their intensified class (genes where both RNA abundance and RPF change in the same direction, but with RPF exceeding the expected RNA-driven change). In anota2seq, such genes would be absorbed into either the translation or abundance class, depending on the statistical thresholds applied. In particular, this conservatism did not appear to penalize anota2seq in the Buffering case, where the target class is itself defined by a compensatory relationship between RNA and RPF changes.

Since AUC rewards any score that separates 'positive' from 'negative' genes regardless of whether the underlying regulatory mechanism is correctly identified, classification performance was assessed separately for anota2seq, deltaTE, and Ribotools against the ground-truth labels. All four tools showed near-perfect specificity for the background class, correctly recovering 98–99% of true background genes with minimal spillover (Figure S2A). As expected, for the three regulated classes, deltaTE and Ribotools produced similar results: translation was recovered at 74-76%, abundance at 84–85%, and buffering at 77%, with the dominant source of error in each case being false negatives rather than confusion between regulated classes, which remained below 2% throughout. Anota2seq failed to recover translation (62%) and, more notably, 21% of true abundance genes were misclassified as translation. The F1 and MCC scores were consistently high for abundance and background, moderate for translation and buffering, and broadly comparable between tools, with deltaTE and Ribotools marginally outperforming anota2seq in translation and abundance classification (Figure S2B).

Benchmark 2

Only Ribotools was included for this benchmark (Supplementary Methods S4 – benchmark 2). The dataset contains two replicates per condition, representing a realistic life science experiment, where having only two biological replicates per condition is common. Although no ground truth is available, we showed in the original comparison between days 1 and 5 (no permutation, FDR < 0.05) that substantial translational

Table S1. Features of TEA tools included in the benchmark. Lang.=Language and version installed. Lic./VCS=Licence/Version Control System. Mnt.=Maintained (at least one substantive code commit or release within approximately the past five years). Doc.=Documentation. Layer class.=Layer classification. Notes (CI=Continuous integration, CLI=Command-line interface).

Tool	Lang.	Install	Lic./ VCS	Mnt.	Doc.	Purpose	E2E/ modular	Layer class.	Input	Notes
Ribotools	Py3.13 R4.5	Bioconda 2.0.0	MIT/ GitHub	✓	✓	Data processing, QC/viz., ORF discovery, TEA	✓	✓	Various	CI. CLI/config-based. Tutorials. Extensive docs. Easy on-boarding.
anota2seq	R4.5	Bioconda 1.32.0	GPL-3/ Bioconductor	✓	✓	TEA	×/✓	✓	Counts	Effect size cut-off. n $\geq$ 3 (2 conditions). Performance $\sim$ normalization and filtering. Bioconductor $\neq$ Bioconda version.
Riborex	R4.4	Bioconda 2.3.4	GPL-3/ GitHub	×	×	TEA	×/×	×	Counts	No FDR/effect size. Deprecated.
deltaTE	R4.5	n/a	n/a GitHub	×	×	TEA	×/×	✓	Counts	Not a package (not installable). No FDR/effect size. Hard-coded parameters, conditions.

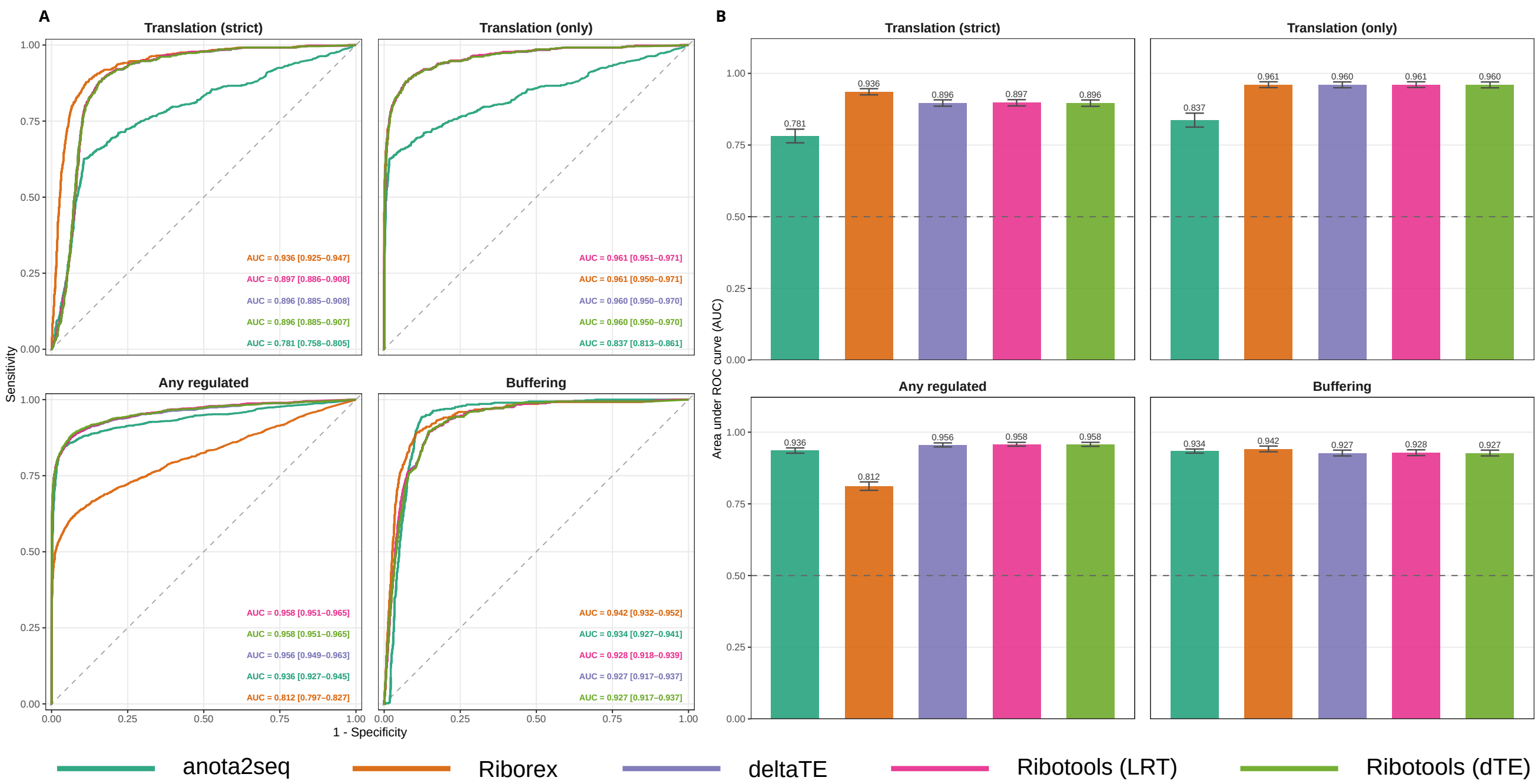

Fig. S1. **A** Benchmark 1. Receiver operating characteristic (ROC) curves for benchmarked tools across four test cases derived from the Oertlin *et al.* simulated dataset [28]: Translation (strict), where true positives are translation genes and true negatives comprise all remaining classes; Translation (only), where true negatives are restricted to background genes; Any regulated, where all regulated genes (translation, buffering, and abundance) are treated as true positives against background; and Buffering, where buffering genes are true positives and all remaining classes are true negatives. The class labels are those from anota2seq (Supplementary Methods S4). Curves are based on gene-level scores (-log10 adjusted p-value). **B** Benchmark 1. Area under the ROC curve (AUC) with 95% confidence intervals (DeLong method) for each tool across the four test cases.

reprogramming was involved, and these observations were consistent with current knowledge of the system, providing a biologically significant positive control for the presence of a

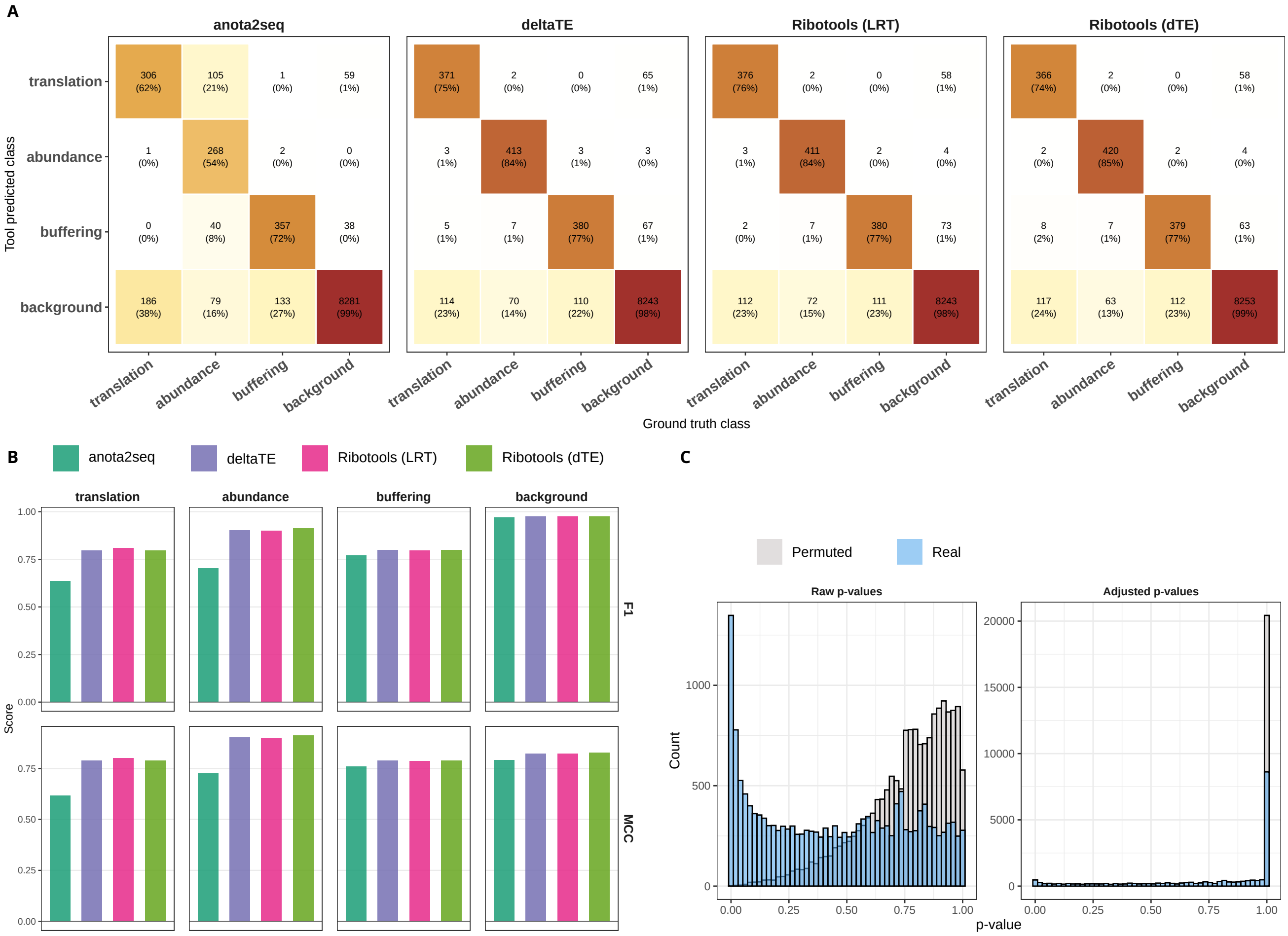

Fig. S2. A Benchmark 1. Confusion matrices comparing predicted regulatory class assignments to the known ground truth labels (translation, buffering, abundance, background) from the Oertlin *et al.* simulated dataset [28]. The number of genes predicted in each class and the corresponding percentage of the true class total are shown in each cell. Cells are colored according to the percentage of the true class correctly predicted, with darker shading indicating higher agreement with the ground truth. Only tools implementing a regulatory layer classification are included. **B** Benchmark 1. Per-class F1 score and Matthews Correlation Coefficient (MCC) for each classifying tool, computed against the ground truth regulatory labels. Only tools implementing a regulatory layer classification are included. **C** Benchmark 2. Distribution of raw (left) and Benjamini-Hochberg adjusted (right) p-values for the translational efficiency (TE) interaction test on the real dataset (blue) and one representative label permutation (gray), shown for Ribotools (LRT).

genuine signal. To assess the false positive rate of our analysis, we performed all four valid label permutations. Under a well-calibrated FDR procedure, the expected proportion of genes tested called significant is controlled at approximately 5%, although this is often much lower. The raw p-value distribution for the TE interaction test in real data was close to uniform, with a modest enrichment at low p-values, consistent with a minority of truly differentially translated genes ($\pi_0 \sim 0.93$, the estimated proportion of the true null hypothesis after BH correction, Figure S2C). With permuted labels, the distribution was instead right-skewed, reflecting the conservative behavior of DESeq2 at $n = 2$ replicates per condition [26]. Correspondingly, zero genes reached FDR < 0.05 in any of the four valid permutations, whereas 907 of 16,946 genes tested for TE interaction alone were significant in real data. Since the same procedure yielded no hits under the permuted null (by construction, all genes must be false positives), this supports the specificity of the 907 genes identified as differentially translated in real data.

Results S2. Qualitative comparison of ORF discovery tools

ORF discovery can be defined as the experimental and computational process of identifying putative protein-coding regions or, more generally, translated regions that do not necessarily depend on the properties of their products or their sequence. These regions are generally referred to as 'Ribo-seq ORFs', especially when the evidence is based on ribosome profiling experiments, or as 'translons' in a general case [37]. A curated list of published ORF discovery tools is given in Table S2. This table presents a light, qualitative and up-to-date overview rather than an exhaustive summary; for in-depth comparisons and methodology breakdowns, we refer readers to dedicated reviews on the subject [42, 22, 41].

Qualifying criteria

Only established tools that showed evidence of active or recent maintenance were included. QC-only or online-only tools were excluded, for example, Trips-Viz, an interactive platform within the RiboSeq.Org ecosystem that supports a range of

analyses (including differential gene expression), but relies on heuristic, visual cues, such as frame-colored read-density and periodicity plots, to highlight translation events [39]. RiboSeq.Org as a whole (which also comprises GWIPS-viz, a genome browser, RiboGalaxy, mentioned in Supplementary Methods S4 – benchmark 1, and the RiboSeq Data Portal, a centralized dataset access) addresses important challenges in data reuse and reproducibility and constitutes a valuable resource. However, it does not provide 'translated ORF calls' as an output, and does not implement a standalone, rigorous ORF discovery pipeline. We also excluded general Ribo-seq data analysis tools such as riboviz2 [8], an E2E Nextflow pipeline for data processing and visualization, but not an ORF discovery tool *per se*. ORF-RATER [14], RiboTaper [4], riboHMM [33], RiboORF2 [17], and SPECtre [6] were all considered deprecated. They provide no installable package, depend on outdated dependencies, or are marked obsolete by the authors themselves. We did not include DeepRibo [7], a supervised ORF discovery tool only for prokaryotes.

Ribotools stands out as the best all-around performer

Ribotools, including Rp-Bp, is the only fully modular, command-line interface (CLI), configuration-based E2E workflow of its kind. The pipeline is designed to process data from raw FASTQ files through the final identification of Ribo-seq ORFs and TEA results. At the same time, analyses can be initiated or repeated from any stage of the workflow, and the pipeline accepts standard input formats generated by other software, including partially processed reads, alignment files, and read-count tables. ORFik's API provides composable functions, but no single pipeline. While it does provide E2E functionality, its modularity remains limited, *e.g.* the main TEA function requires an ORFik experiment class, built around file system paths, and there is no lightweight stub constructor designed for this use case. PRICE also provides E2E functionality; however, we were unable to determine whether it was actively maintained. It is part of a larger ecosystem, named GEDI, which includes modules to pre-process, map reads, and quantify actively translated codons and ORFs. This java-based framework has the advantage of being platform-independent, but its installation is not trivial. Its use of the CIT file format can break interoperability. All other tools considered only handle alignment (BAM) files, with the exception of ORFquant, which also accepts the output of Ribo-seQC, but otherwise provides limited functionality. The ORFquant package is only installable via the source repository, and except for minor updates in 2023, we were unable to find any commits or releases after 2020. Issues and pull-requests (PRs) remain unaddressed. RiboCode's last release dates from 2021, and there is no evidence of code maintenance since 2022. Open issues and PRs also remain unanswered. In addition to Ribotools, ORFik and PRICE can handle *de novo* ORF calling; but we were unable to find direct evidence, documentation, vignette, or published examples demonstrating this capability for other tools. Ribotools and Rp-Bp each provide extensive documentation with detailed tutorials and test dataset, facilitating on-boarding. ORFik's documentation is organized into vignettes, but its usage is not trivial (entry-level to intermediate R scripting skills required). The package is under active development and provides additional functionality for CAGE-, TCP-seq or RCP-seq data, for automatic download of genome annotation, and visualization (static plots only). Ribo-TISH is one of the only software that provides functionality for mapping and analyzing translation initiating sites (TISs), but its overall scope remains limited. The PRICE documentation is only organized into a GitHub Wiki, but not hosted on a dedicated documentation service. A genome browser (GB) is available, but otherwise there is no general purpose quality control or visualization functionality. Ribotools does not yet provide interactive visualization for TEA results, but includes with Rp-Bp two lightweight web applications for Ribo-seq quality control and ORF discovery. ORFik predicts Ribo-seq ORFs using a rule-based approach and metrics such as ORFscore or FLOSS. Ribotricer uses a phase score, but choosing the right phase score and coverage cutoff might not be trivial (can be learned if RNA-seq data are also available). Rp-Bp is fully unsupervised, performs automatic P-site adjustment per read length, and annotate ORFs according to a standardized terminology, however it does not handle overlapping ORFs, something that PRICE can do.

Results S3. Transcriptome and translatome of hiPSCs differentiation into cardiomyocytes

Providing functional or protein-level evidence for non-canonical translation events at scale requires substantial resources that are often only available through large collaborative consortia [10]. Such validation is therefore beyond the scope of the present work. Instead, our aim is to demonstrate how Ribotools, together with Rp-Bp, facilitates ORF discovery at an early stage by generating a ready-to-use catalogue of annotated Ribo-seq ORFs in BED6, extended BED12, and FASTA (nucleotide and amino acid sequence) formats. Consistent with previous studies, a large proportion of sORFs and the majority of uORFs had been reported previously (manuscript Fig. 2F). However, approximately 72% of the ncORFs identified in our analysis appear to represent previously unreported translation events. Although uORFs constitute a substantial fraction of all sORFs, and many ncORFs are also shorter than 100 amino acids, only the longer ORFs were supported by mass spectrometry (MS) evidence (Supplementary Figure S4A). We also found 259 sORFs with a significant p value (<0.1) according to RNAcode, 439 sORFs with a positive deciban score according to PhyloCSF, and 1701 sORFs with at least one InterPro domain annotation or signature (Supplementary Methods S5 and Figure S4B). These results support the biological relevance of non-canonical Ribo-seq ORFs, with a subset being supported by independent lines of evidence, including evolutionary conservation, protein domain annotation, and, to a lesser extent, mass spectrometry.

Table S2. Features of ORF discovery tools. Lic./VCS=Licence/Version Control System. Install=Package installation (Bioc.=Bioconductor. Conda=Bioconda incl. containers. GitHub=Developer install. PyPI=Python Package Index - with limited functionality). Mnt.=Maintained (at least one substantive code commit or release within approximately the past five years). Doc.=Documentation (static hosted, developer hub, *etc.*, beyond a 'README page'). Features=Special features in addition to ORF discovery (Trim.=Adapter trimming/quality filtering. Align.=Align reads incl. contaminant removal such as ribosomal RNA. P-site auto.:Automatic P-site shifting. QC=Quality control. Trx. filt.=Transcript filtering. TIS detec.=Translation initiation site detection. diff TIS=Differential TIS analysis). Viz.=Visualization (interactive). E2E=End-to-end (from raw reads to end product). Model=Periodicity and/or translation prediction estimation (Unsuper.=Unsupervised). ORF call.=*de novo* ORF calling (accepts a *de novo*-assembled, non-reference transcriptome as input and labels, in some form or other, resulting calls as 'novel').

Tool	Lic./ VCS	Install	Mnt.	Doc.	Features	Viz.	E2E	Model	ORF call.	Notes
Ribotools	MIT GitHub	Conda PyPI GitHub	✓	✓	Trim., Align., P-site auto., QC, TEA	✓	✓	Unsuper. Bayesian	✓	Incl. Rp-Bp. CLI. Config-based. Annotate ORFs.
ORFik	MIT GitHub	Bioc. Conda GitHub	✓	✓	Trim., Align., P-site auto., QC, TEA	✓	✓	Heuristic	✓	Composable R code, no pipeline. CAGE-, TCP-, RCP-seq. ORFik experiment
ORFquant	GPLv3 GitHub	Github	✓	×	Trx. filt.	×	×	Unsup. Multitapper	✓	Ribo-seQC. BAM. Only AUG
RiboCode	MIT GitHub	Conda PyPI GitHub	✓	×	P-site auto., ORF count.	×	×	Statistical. Prop. test	✓	Transcriptome BAM. CLI.
Ribo-TISH	GPLv3 GitHub	Conda PyPI GitHub	✓	×	QC, TIS/ORF detec., diff. TIS	×	×	Statistical	✓	BAM. CLI.
PRICE	GPLv3 GitHub	n/a	✓	✓	Trim., Align.	✓	✓	Unsuper. EM.	✓	GEDI. Expert install. CIT. GB. Overlapping ORFs
ribotricer	GPLv3 GitHub	Conda PyPI GitHub	✓	×	QC	×	×	Unsuper. Phase score	✓	BAM. Phase-score cutoff

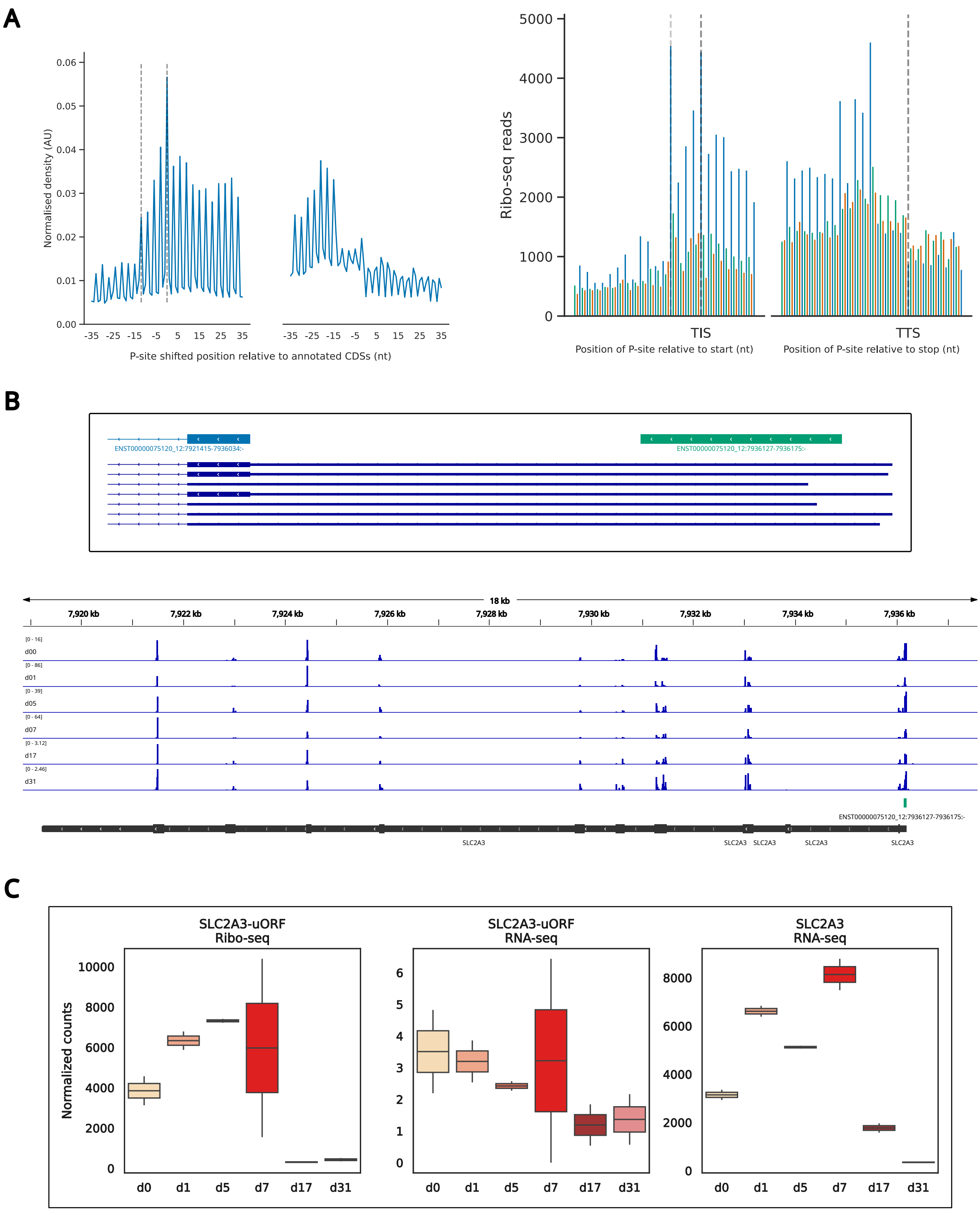

Fig. S3. A Image adapted from the quality control dashboard, available at https://rpbp.dieterichlab.org/hIPSCCMdiff/profile_construction. Global metagene profile for all samples (left). Annotated translation initiation start site (TIS) and -12 offset are indicated by a dashed line. Metagene profile for one sample and read length used for analysis, showing a clear peak at -12 (offset) and a second peak at the TIS, corresponding to the start of the fifth codon (right). **B** Image adapted from the ORF discovery dashboard, available at https://rpbp.dieterichlab.org/hIPSCCMdiff/predictions. Zoom from the IGV browser showing c12riboseqorf16, or SLC2A3-uORF (annotated as ENST00000075120_12:7936127-7936175:- by Rp-Bp), upstream of the glycolysis gene SLC2A3, including compatible transcripts (top). Ribo-seq p-site coverage supporting c12riboseqorf16 (bottom). **C** Box plots with normalized expression levels (Ribo- and RNA-seq) of SLC2A3-uORF, also known as c12riboseqorf16, and of SLC2A3 across developmental stages. Boxes indicate interquartile range; center line indicates median.

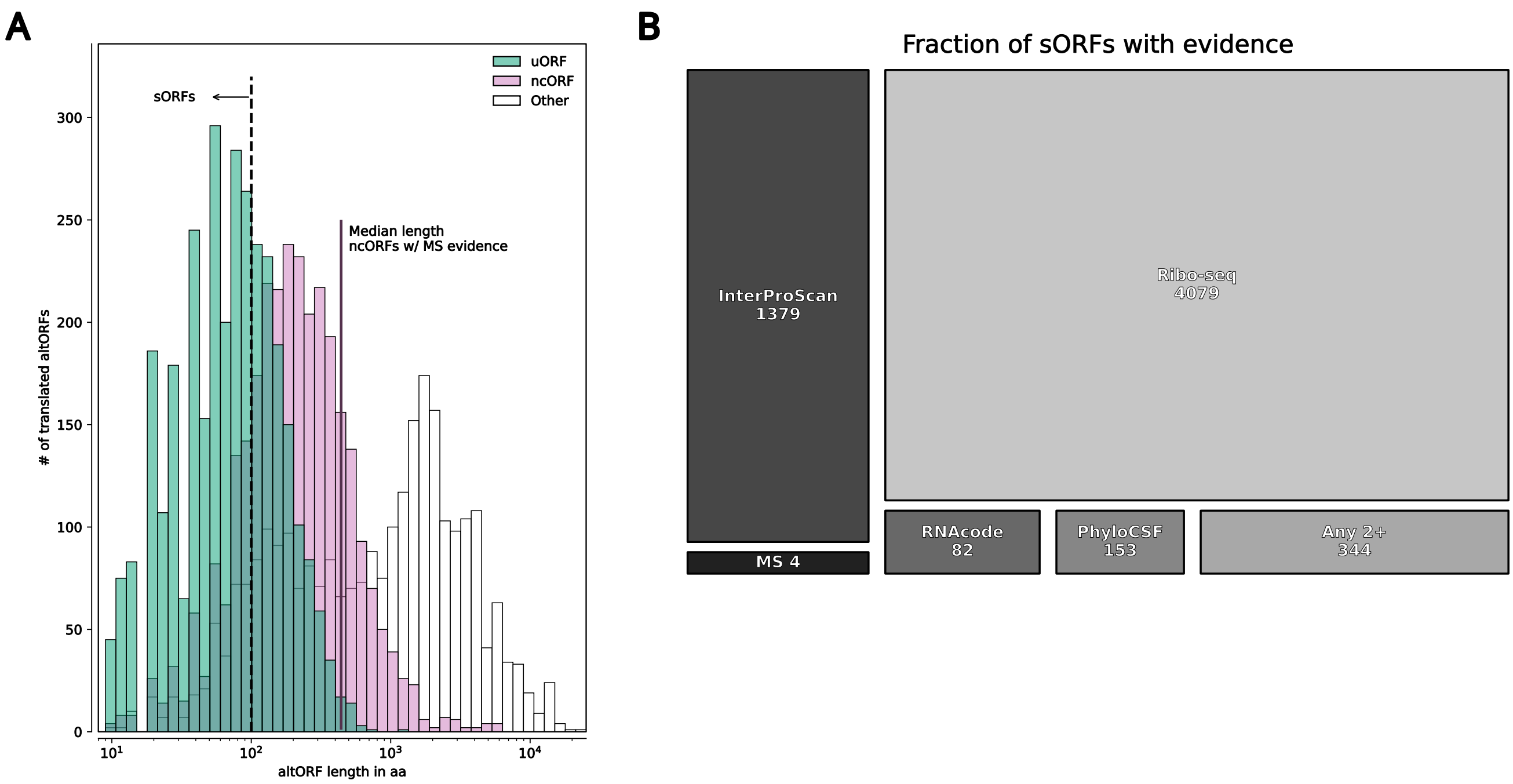

Fig. S4. A Histogram of alternative ORF lengths showing upstream ORFs (uORFs), ORFs from transcripts annotated as non-coding (ncORFs), and other predicted Ribo-seq ORFs (alternative CDS, internal, downstream, or novel ORFs). 76 longer ncORFs are supported by mass spectrometry evidence. **B** Treemap representing the fraction of small ORFs (sORFs) with additional evidence, either from mass spectrometry (MS), InterProScan, RNAcode, PhyloCSF, or a combination of any 2 or more of these.

Results S4. Translational control during hiPSCs differentiation into cardiomyocytes

Protein correlation analysis

Protein fold changes were positively and significantly correlated with both RNA and RPF fold changes for the *forwarded* and *intensified* classes (R = 0.68–0.83, Figure S8), consistent with these genes being regulated concordantly, with both axes well-powered. *Buffered* genes generally showed a positive correlation with protein, comparable to or lower than *forwarded* genes, and consistently higher for RNA than for RPF (R = 0.46–0.72 for RNA, R = -0.3–0.5 for RPF). This class combines two components: genes with active counter-regulation, where both RNA and RPF are significant but act in opposite directions, and genes where only RNA is significant, compensated by a TE change (*i.e.* only the RNA axis is well-powered). The latter component thus contributes with negligible, non-significant RPF fold changes (Wald test), but significant TE (not visible), which may manifest as an anti-correlation between protein and RPF (Supplementary Figure S5B and S8F, day 31 vs. day 17).

The *exclusive* class is defined by a significant Ribo-seq change and a significant interaction (TE) term, but without a corresponding significant RNA change, *i.e.* only the RPF axis is well-powered. These genes generally showed a weaker but still positive and significant correlation with protein, with one clear exception in the d5 vs d1 comparison. A non-significant RNA term does not necessarily imply zero effect for this class: at n = 2 replicates per condition, many genes with a real, moderate RNA change may fail to reach significance from lack of power rather than a true absence of transcriptional regulation, and the correlation with protein can aggregate this signal across genes even where individual tests are underpowered – a more direct effect than in the *buffered* case, which combines two distinct modes of regulation. It is also worth noting that this class is defined by a significant interaction term (LRT p-value), whereas the direction assigned to 'the translation effect' is taken from a separate RPF Wald model. Since RPF reflects ribosome occupancy, a snapshot of translation at the sampled time point, while protein abundance is the time-integrated net balance of synthesis and degradation, an immediate, detectable protein-level change is not always expected, particularly for exclusive genes whose translational response may not yet be reflected at the protein level. Overall, these results are broadly concordant with the expectations of the Ribotools classification, supporting it as a meaningful representation of the translational dynamics experienced by these cells.

Contrast	Layer	GO Biological Process	Reactome
d5 vs d0	Exclusive	**GO:0034242** Neg. reg. syncytium formation (−1.7)	**R-HSA-72613** Euk. transl. initiation (−2.1)
		–	**R-HSA-156842** Euk. transl. elongation (−2.2)
		–	**R-HSA-927802** Nonsense-mediated decay (−2.0)
		–	**R-HSA-9633012** Response of EIF2AK4/GCN2 (−2.0)
	Buffered	**GO:0060411** Cardiac septum morphogen. (2.3)	**R-HSA-9830369** Kidney development (2.0)
		**GO:0055008** Cardiac muscle morphogen. (2.2)	–
		**GO:0003203** Endocardial morphogenesis (2.1)	–
		**GO:0035270** Endocrine system dev. (2.0)	–
d5 vs d1	Exclusive	**GO:0002181** Cytoplasmic translation (−2.2)	**R-HSA-72312** rRNA processing (−2.8)
		**GO:0034242** Neg. reg. syncytium formation (−2.2)	**R-HSA-9633012** Response of EIF2AK4/GCN2 (−2.6)
		**GO:0060142** Reg. of syncytium formation (−2.0)	**R-HSA-156842** Euk. transl. elongation (−2.6)
		**GO:0006949** Syncytium formation (−1.7)	**R-HSA-72613** Euk. transl. initiation (−2.6)
d7 vs d5	Exclusive	**GO:0051307** Meotic chrom. separation (−2.0)	–
		–	–
		–	–
		–	–
	Forwarded	**GO:0003015** Heart process (2.2)	–
		**GO:0008016** Reg. heart contraction (2.2)	–
		**GO:0006936** Muscle contraction (2.2)	–
		**GO:0048738** Cardiac muscle dev. (2.2)	–
d17 vs d7	Buffered	**GO:0034242** Neg. reg. syncytium formation (−2.1)	–
		–	–
		–	–
		–	–
d31 vs d17	Buffered	**GO:0042060** Wound healing (−1.8)	**R-HSA-1474244** Extracell. matrix organization (−1.8)
		**GO:0001501** Skeletal system dev. (−1.8)	**R-HSA-9006934** Signalling by RTKs (−1.6
		**GO:0030855** Epithelial cell diff. (−1.7)	–
		**GO:0003012** Muscle system process (1.5)	–

Table S3. Gene set enrichment analysis (GSEA). Top four enriched Gene Ontology (GO) Biological Process and Reactome pathways per developmental stage and regulatory layer. Values in parentheses indicate the normalized enrichment score (NES). Missing contrasts and/or layers indicate that no significant pathway enrichment was found. For contrasts and/or layers with at least one significant pathway enrichment, dashes indicate that fewer than four significant pathways were identified.

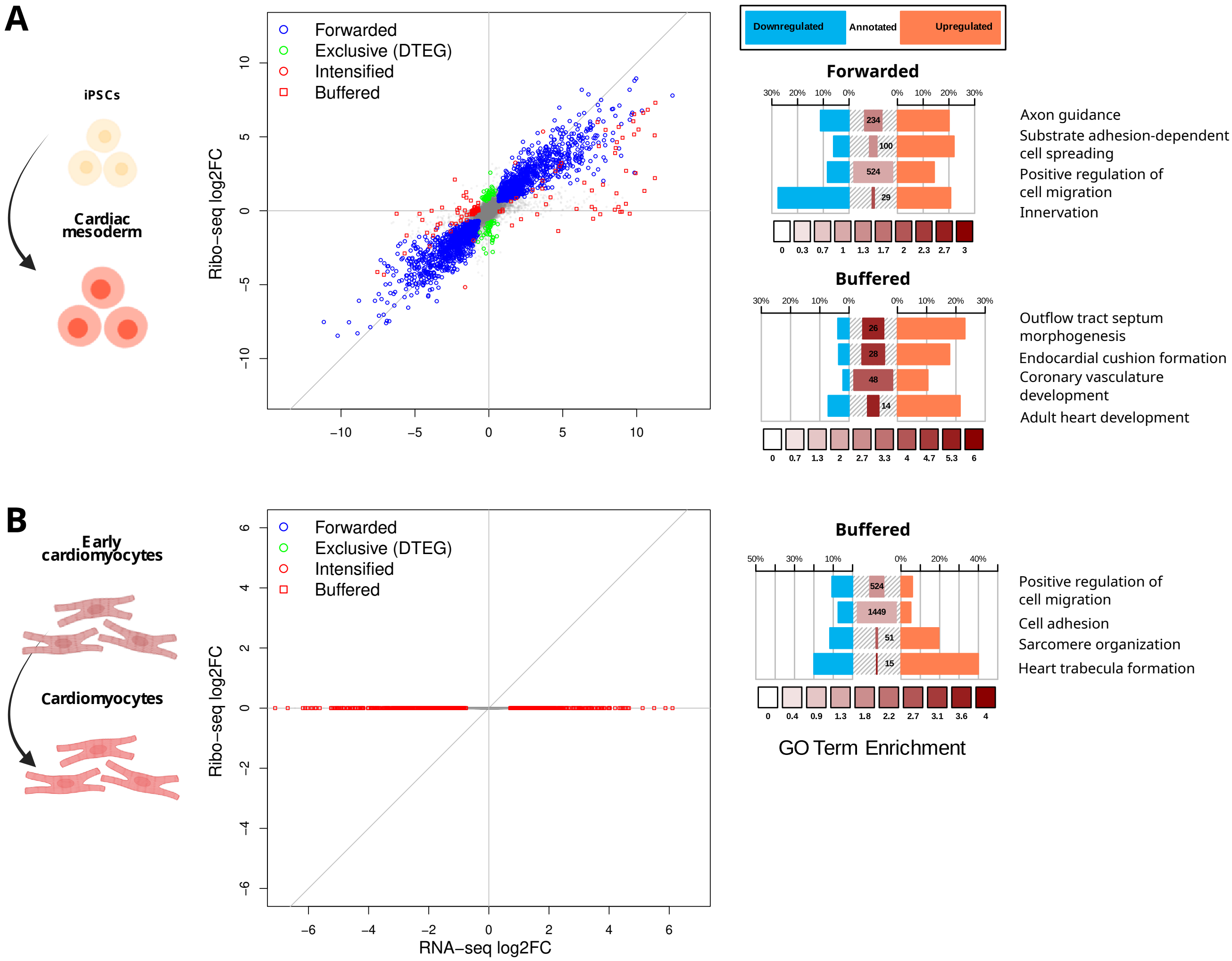

Fig. S5. Transcription and translation are highly dynamic during differentiation. The comparisons show **A** day 5 vs. day 0 (from iPSCs to cardiac mesoderm), and **B** day 31 vs. day 17 (from early to mature CMs). Scatter plots showing the output of Ribotools are shown in the middle. Gene ontology (Biological Process) over-representation analyses for selected classes (forwarded, exclusive, buffered, or intensified), as determined by Ribotools, are shown on the right.

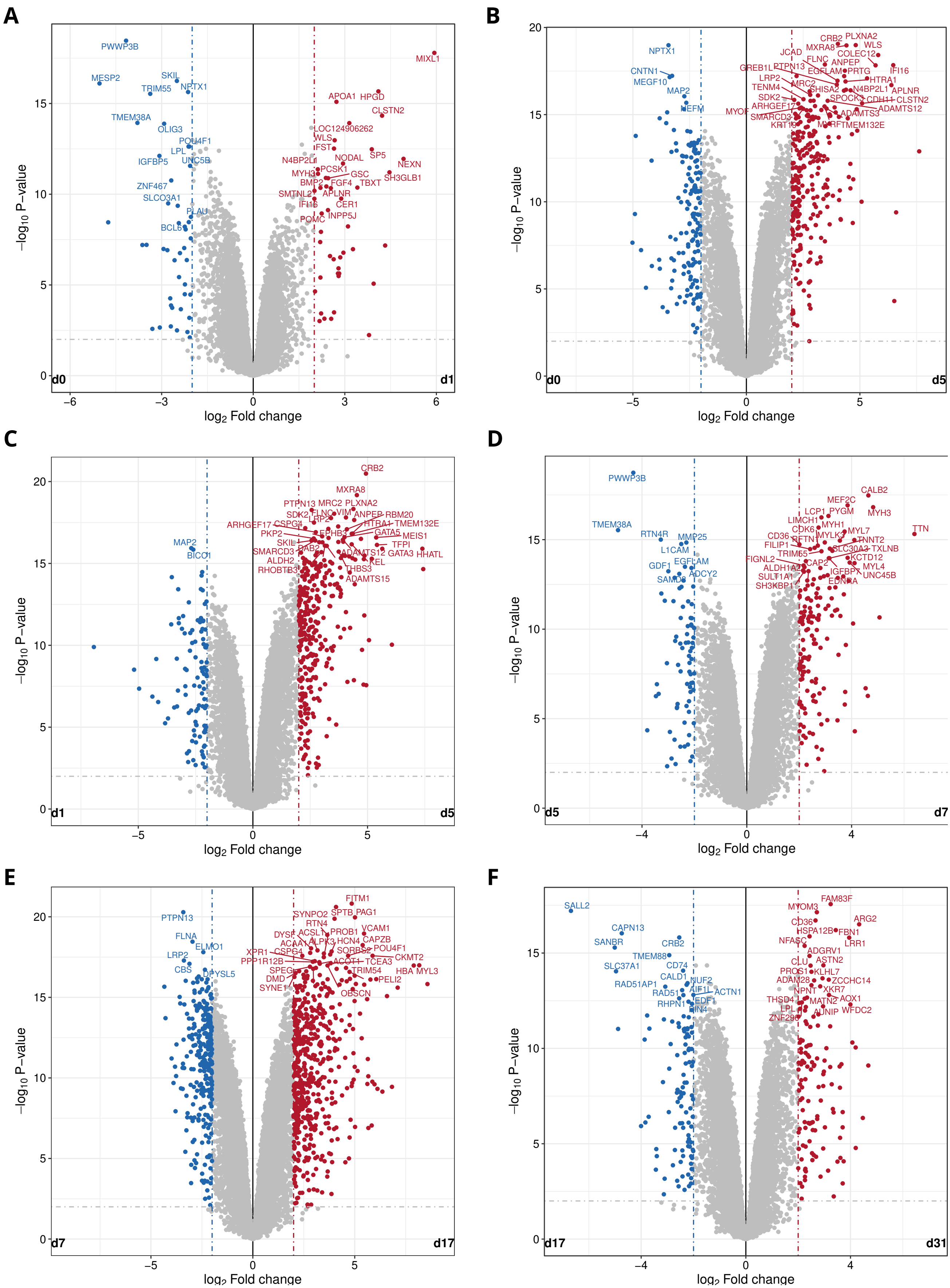

Fig. S6. Differential proteomics. Volcano plots showing comparisons for **A** day 1 vs. day 0, **B** day 5 vs. day 0, **C** day 5 vs. day 1, **D** day 7 vs. day 5, **E** day 17 vs. day 7, and **F** day 31 vs. day 17. Proteins were tested at a significance threshold (adjusted p-value) of 0.01, log2 fold change threshold of 2, and FDR were reported after Benjamini-Hochberg correction.

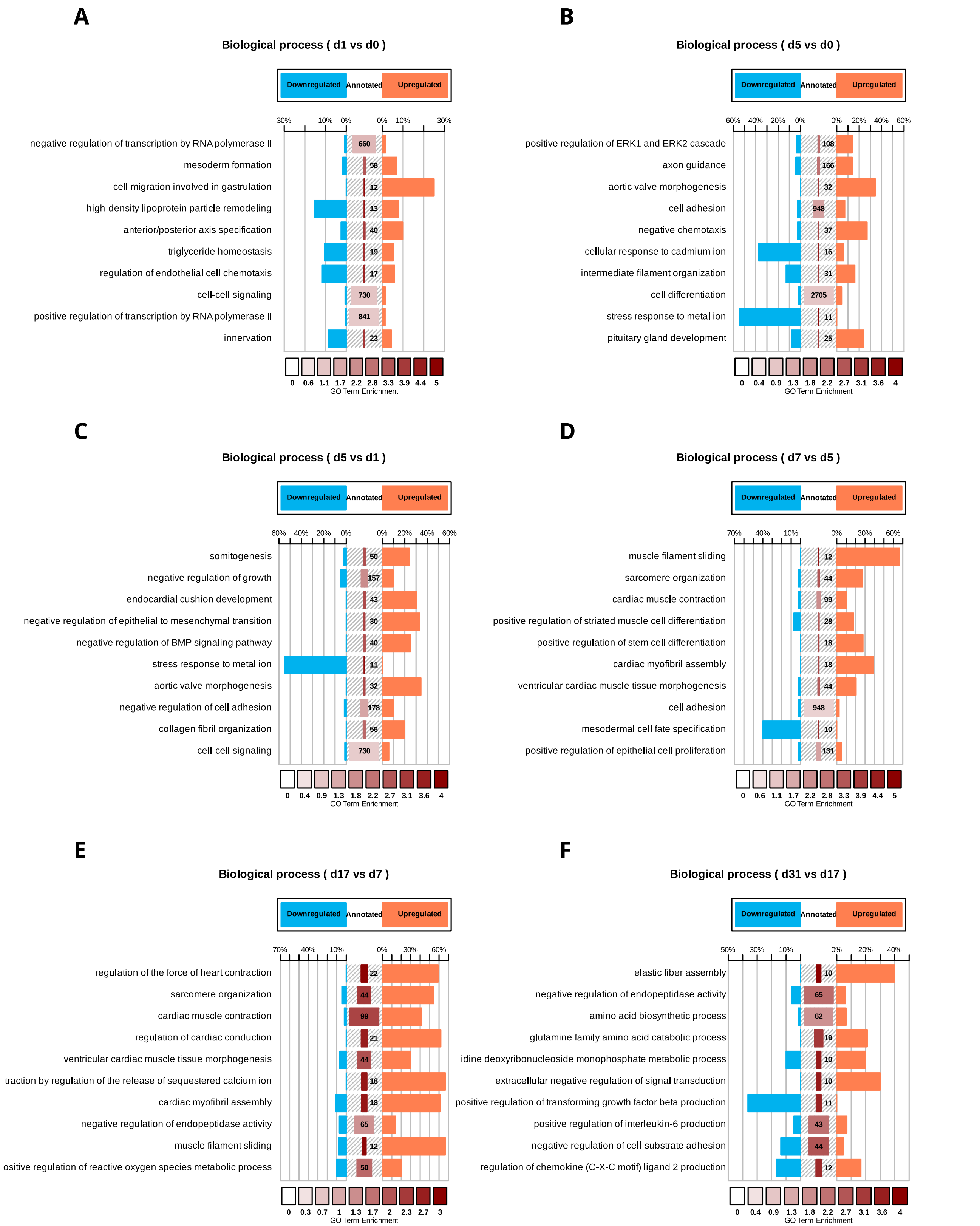

Fig. S7. Gene ontology (Biological Process) over-representation analysis for the differentially expressed proteins shown in Fig. S6. Bar plots showing comparisons for **A** day 1 vs. day 0, **B** day 5 vs. day 0, **C** day 5 vs. day 1, **D** day 7 vs. day 5, **E** day 17 vs. day 7, and **F** day 31 vs. day 17.

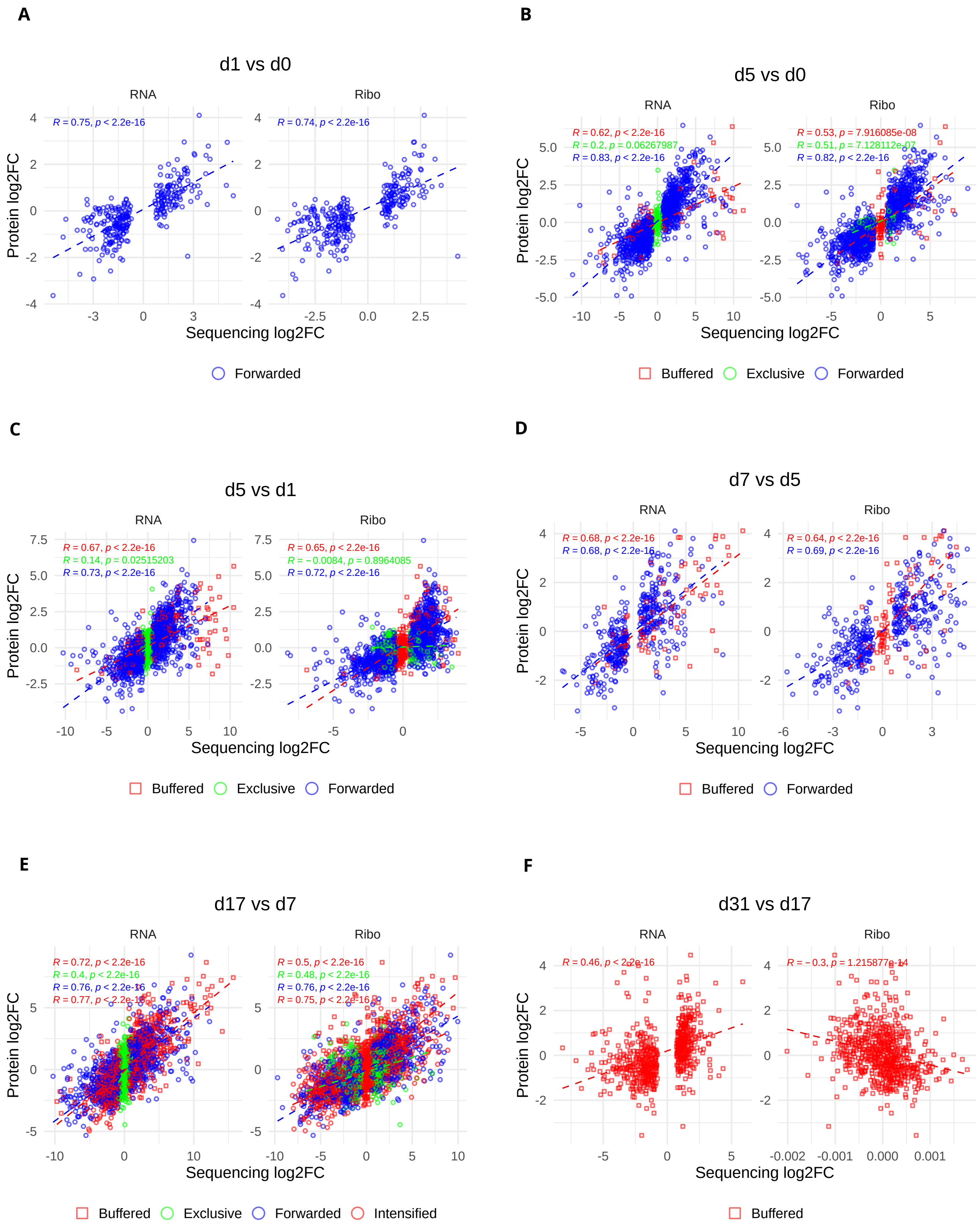

Fig. S8. Concordance between sequencing-based (RNA- and Ribo-seq) and protein fold changes. Scatter plots showing comparisons for **A** day 1 vs. day 0, **B** day 5 vs. day 0, **C** day 5 vs. day 1, **D** day 7 vs. day 5, **E** day 17 vs. day 7, and **F** day 31 vs. day 17. For each transition, the left and right scatter plots compare proteomics log2 fold changes with RNA-seq and Ribo-seq log2 fold changes, respectively. Colors and symbols indicate Ribotools regulatory classes. Spearman's R and associated p-value are reported for each class within each panel. Not all classes are present in all contrasts, reflecting differences in the number of genes assigned to each regulatory mode at each stage (*cf.* Figure 3 and Supplementary Figure S5).

References

1. A Alexa and J Rahnenfuhrer. *topGO: Enrichment Analysis for Gene Ontology*, 2018. R package version 2.28.0.
2. Jordan A. Berg, Jonathan R. Belyeu, Jeffrey T. Morgan, Yeyun Ouyang, Alex J. Bott, Aaron R. Quinlan, Jason Gertz, and Jared Rutter. Xpressyourself: Enhancing, standardizing, and automating ribosome profiling computational analyses yields improved insight into data. *PLOS Computational Biology*, 16(1):1–20, 01 2020.
3. E. Boileau. Rp-bp and the ribotools box, 2026.
4. Lorenzo Calviello, Neelanjan Mukherjee, Emanuel Wyler, Henrik Zauber, Antje Hirsekorn, Matthias Selbach, Markus Landthaler, Benedikt Obermayer, and Uwe Ohler. Detecting actively translated open reading frames in ribosome profiling data. *Nature Methods*, 13(2):165–170, Feb 2016.
5. Sonia Chothani, Eleonora Adami, John F. Ouyang, Sivakumar Viswanathan, Norbert Hubner, Stuart A. Cook, Sebastian Schafer, and Owen J. L. Rackham. deltate: Detection of translationally regulated genes by integrative analysis of ribo-seq and rna-seq data. *Current Protocols in Molecular Biology*, 129(1):e108, 2019.
6. Sang Y. Chun, Caitlin M. Rodriguez, Peter K. Todd, and Ryan E. Mills. Spectre: a spectral coherence-based classifier of actively translated transcripts from ribosome profiling sequence data. *BMC Bioinformatics*, 17(1):482, Nov 2016.
7. Jim Clauwaert, Gerben Menschaert, and Willem Waegeman. Deepribo: a neural network for precise gene annotation of prokaryotes by combining ribosome profiling signal and binding site patterns. *Nucleic Acids Research*, 47(6):e36–e36, 04 2019.
8. Alexander L Cope, Felicity Anderson, John Favate, Michael Jackson, Amanda Mok, Anna Kurowska, Junchen Liu, Emma MacKenzie, Vikram Shivakumar, Peter Tilton, Sophie M Winterbourne, Siyin Xue, Kostas Kavoussanakis, Liana F Lareau, Premal Shah, and Edward W J Wallace. riboviz 2: a flexible and robust ribosome profiling data analysis and visualization workflow. *Bioinformatics*, 38(8):2358–2360, 04 2022.
9. Petr Danecek, James K Bonfield, Jennifer Liddle, John Marshall, Valeriu Ohan, Martin O Pollard, Andrew Whitwham, Thomas Keane, Shane A McCarthy, Robert M Davies, and Heng Li. Twelve years of SAMtools and BCFtools. *GigaScience*, 10(2), 02 2021. giab008.
10. Eric W. Deutsch, Leron W. Kok, Jonathan M. Mudge, Cristian F. Valls, Irwin Jungreis, Jorge Ruiz-Orera, Zhi Sun, Ulrike Kusebauch, Ivo Fierro-Monti, Jennifer G. Abelin, M. Mar Alba, Julie L. Aspden, Sreejan Bandyopadhyay, Kaushik Banerjee, Pavel V. Baranov, and et al. Expanding the human proteome with microproteins and peptideins. *Nature*, 654(8119):813–825, Jun 2026.
11. A Dobin, CA Davis, F Schlesinger, J Drenkow, C Zaleski, S Jha, P Batut, M Chaisson, and TR Gingeras. Star: ultrafast universal rna-seq aligner. *Bioinformatics*, 29(1):15–21, 2013.
12. M Dodt, JT Roehr, R Ahmed, and C Dieterich. Flexbar – flexible barcode and adapter processing for next-generation sequencing platforms. *Biology*, 1(3):895–905, 2012.
13. Alla D. Fedorova, Jack A.S. Tierney, Audrey M. Michel, and Pavel V. Baranov. Ribogalaxy: A galaxy-based web platform for ribosome profiling data processing – 2023 update. *Journal of Molecular Biology*, 435(14):168043, 2023. Computation Resources for Molecular Biology.
14. Alexander P. Fields, Edwin H. Rodriguez, Marko Jovanovic, Noam Stern-Ginossar, Brian J. Haas, Philipp Mertins, Raktima Raychowdhury, Nir Hacohen, Steven A. Carr, Nicholas T. Ingolia, Aviv Regev, and Jonathan S. Weissman. A regression-based analysis of ribosome-profiling data reveals a conserved complexity to mammalian translation. *Molecular Cell*, 60(5):816–827, 2015.
15. Björn Grüning, Ryan Dale, Andreas Sjödin, Brad A. Chapman, Jillian Rowe, Christopher H. Tomkins-Tinch, Renan Valieris, Johannes Köster, and The Bioconda Team. Bioconda: sustainable and comprehensive software distribution for the life sciences. *Nature Methods*, 15(7):475–476, Jul 2018.
16. Storey J, Bass A, Dabney A, and Robinson D. *qvalue: Q-value estimation for false discovery rate control*, 2026. R package version 2.44.0.
17. Zhe Ji. Riborf: Identifying genome-wide translated open reading frames using ribosome profiling. *Current Protocols in Molecular Biology*, 124(1):e67, 2018.
18. Johannes Köster and Sven Rahmann. Snakemake—a scalable bioinformatics workflow engine. *Bioinformatics*, 28(19):2520–2522, 10 2012.
19. B Langmead and SL Salzberg. Fast gapped-read alignment with bowtie 2. *Nature Methods*, 9(4):357–359, 2012. PMID: 36599300.
20. Rachel Legendre, Agnès Baudin-Baillieu, Isabelle Hatin, and Olivier Namy. Ribotools: a galaxy toolbox for qualitative ribosome profiling analysis. *Bioinformatics*, 31(15):2586–2588, 08 2015.
21. Keren Li, C Matthew Hope, Xiaozhong A Wang, and Ji-Ping Wang. Ribodipa: a novel tool for differential pattern analysis in ribo-seq data. *Nucleic Acids Research*, 48(21):12016–12029, 12 2020.
22. Mingso Sherma Limbu, Tianze Xiong, and Sufang Wang. A review of ribosome profiling and tools used in ribo-seq data analysis. *Computational and Structural Biotechnology Journal*, 23:1912–1918, 2024.
23. Michael F. Lin, Irwin Jungreis, and Manolis Kellis. Phylocsf: a comparative genomics method to distinguish protein coding and non-coding regions. *Bioinformatics*, 27(13):i275–i282, 07 2011.
24. Qi Liu, Tanya Shvarts, Piotr Sliz, and Richard I Gregory. Ribotoolkit: an integrated platform for analysis and annotation of ribosome profiling data to decode mrna translation at codon resolution. *Nucleic Acids Research*, 48(W1):W218–W229, 07 2020.
25. Fabricio Loayza-Puch, Koos Rooijers, Levi C. M. Buil, Jelle Zijlstra, Joachim F. Oude Vrielink, Rui Lopes, Alejandro Pineiro Ugalde, Pieter van Breugel, Ingrid Hofland, Jelle Wesseling, Olaf van Tellingen, Axel Bex, and Reuven Agami. Tumour-specific proline vulnerability uncovered by differential ribosome codon reading. *Nature*, 530(7591):490–494, Feb 2016.
26. Michael I. Love, Wolfgang Huber, and Simon Anders. Moderated estimation of fold change and dispersion for rna-seq data with deseq2. *Genome Biology*, 15(12):550, Dec 2014.
27. Brandon Malone, Ilian Atanassov, Florian Aeschimann, Xinping Li, Helge Großhans, and Christoph Dieterich. Bayesian prediction of RNA translation from ribosome profiling. *Nucleic Acids Research*, 45(6):2960–2972, 01 2017. PMID: 28126919.

28. Christian Oertlin, Julie Lorent, Carl Murie, Luc Furic, Ivan Topisirovic, and Ola Larsson. Generally applicable transcriptome-wide analysis of translation using anota2seq. *Nucleic Acids Research*, 47(12):e70–e70, 03 2019.
29. Adam B. Olshen, Andrew C. Hsieh, Craig R. Stumpf, Richard A. Olshen, Davide Ruggero, and Barry S. Taylor. Assessing gene-level translational control from ribosome profiling. *Bioinformatics*, 29(23):2995–3002, 12 2013.
30. Geo M. Pertea and Mihaela Pertea. Gff utilities: Gffread and gffcompare [version 2; peer review: 3 approved]. *F1000Research*, 9(304):1–20, 2020.
31. Mihaela Pertea, Geo M. Pertea, Corina M. Antonescu, Tsung-Cheng Chang, Joshua T. Mendell, and Steven L. Salzberg. Stringtie enables improved reconstruction of a transcriptome from rna-seq reads. *Nature Biotechnology*, 33(3):290–295, Mar 2015.
32. Givanna H Putri, Simon Anders, Paul Theodor Pyl, John E Pimanda, and Fabio Zanini. Analysing high-throughput sequencing data in python with htseq 2.0. *Bioinformatics*, 38(10):2943–2945, 03 2022.
33. Anil Raj, Sidney H Wang, Heejung Shim, Arbel Harpak, Yang I Li, Brett Engelmann, Matthew Stephens, Yoav Gilad, and Jonathan K Pritchard. Thousands of novel translated open reading frames in humans inferred by ribosome footprint profiling. *eLife*, 5:e13328, may 2016.
34. Juri Rappsilber, Matthias Mann, and Yasushi Ishihama. Protocol for micro-purification, enrichment, pre-fractionation and storage of peptides for proteomics using stagetips. *Nature Protocols*, 2(8):1896–1906, Aug 2007.
35. Xavier Robin, Natacha Turck, Alexandre Hainard, Natalia Tiberti, Frédérique Lisacek, Jean-Charles Sanchez, and Markus Müller. proc: an open-source package for r and s+ to analyze and compare roc curves. *BMC Bioinformatics*, 12(1):77, Mar 2011.
36. Matthew Stephens, Peter Carbonetto, David Gerard, Mengyin Lu, Lei Sun, Jason Willwerscheid, and Nan Xiao. *ashr: Methods for Adaptive Shrinkage, using Empirical Bayes*, 2023. R package version 2.2-63.
37. Michał I. Świrski, Jack A. S. Tierney, M. Mar Albà, Dmitry E. Andreev, Julie L. Aspden, John F. Atkins, Michal Bassani-Sternberg, Marla J. Berry, Stefano Biffo, Kathleen Boris-Lawrie, Mark Borodovsky, and et al. Translon: a single term for translated regions. *Nature Methods*, 22(10):2002–2006, Oct 2025.
38. Toma Tebaldi, Erik Dassi, Galena Kostoska, Gabriella Viero, and Alessandro Quattrone. translatome: an r/bioconductor package to portray translational control. *Bioinformatics*, 30(2):289–291, 01 2014.
39. Jack A S Tierney, Michał I Świrski, Håkon Tjeldnes, Anmol M Kiran, Gionmattia Carancini, Stephen J Kiniry, Audrey M Michel, Joanna Kufel, Eivind Valen, and Pavel V Baranov. Riboseq.org: an integrated suite of resources for ribosome profiling data analysis and visualization. *Nucleic Acids Research*, 53(D1):D268–D274, 01 2025.
40. Håkon Tjeldnes, Kornel Labun, Yamila Torres Cleuren, Katarzyna Chyżyńska, Michał Świrski, and Eivind Valen. Orfik: a comprehensive r toolkit for the analysis of translation. *BMC Bioinformatics*, 22(1):336, Jun 2021.
41. Gregory Tong, Nasun Hah, and Thomas F Martinez. Comparison of software packages for detecting unannotated translated small open reading frames by ribo-seq. *Briefings in Bioinformatics*, 25(4):bbae268, 07 2024.
42. Hongwei Wang, Yan Wang, and Zhi Xie. Computational resources for ribosome profiling: from database to Web server and software. *Briefings in Bioinformatics*, 20(1):144–155, 08 2017. PMID: 28968766.
43. S Washietl, S Findeiss, SA Müller, S Kalkhof, M von Bergen, IL Hofacker, PF Stadler, and N Goldman. Rnacode: robust discrimination of coding and noncoding regions in comparative sequence data. *RNA*, 17(4):578–94, 2011.
44. Hadley Wickham. *ggplot2: Elegant Graphics for Data Analysis*. Springer-Verlag New York, 2016.
45. Tianzhi Wu, Erqiang Hu, Shuangbin Xu, Meijun Chen, Pingfan Guo, Zehan Dai, Tingze Feng, Lang Zhou, Wenli Tang, Li Zhan, Xiaocong Fu, Shanshan Liu, Xiaochen Bo, and Guangchuang Yu. clusterprofiler 4.0: A universal enrichment tool for interpreting omics data. *The Innovation*, 2(3):100141, 2021.
46. Zhengtao Xiao, Qin Zou, Yu Liu, and Xuerui Yang. Genome-wide assessment of differential translations with ribosome profiling data. *Nature Communications*, 7(1):11194, Apr 2016.
47. Zhiyu Xu, Long Hu, Binbin Shi, SiSi Geng, Longchen Xu, Dong Wang, and Zhi J Lu. Ribosome elongating footprints denoised by wavelet transform comprehensively characterize dynamic cellular translation events. *Nucleic Acids Research*, 46(18):e109–e109, 10 2018.
48. Yi Zhong, Theofanis Karaletsos, Philipp Drewe, Vipin T Sreedharan, David Kuo, Kamini Singh, Hans-Guido Wendel, and Gunnar Rätsch. Ribodiff: detecting changes of mrna translation efficiency from ribosome footprints. *Bioinformatics*, 33(1):139–141, 01 2017.
49. Anqi Zhu, Joseph G. Ibrahim, and Michael I. Love. Heavy-tailed prior distributions for sequence count data: removing the noise and preserving large differences. *Bioinformatics*, 2018.
